# MiR‐940 Suppresses Ferroptosis by Controlling Expression of Key Regulatory Genes

**DOI:** 10.1002/advs.75830

**Published:** 2026-05-29

**Authors:** Andrea Kolak, Juliane Tschuck, Stefanie A. I. Weiß, Daniel Kaemena, Karolin Klimm, Ana Galhoz, Larissa Ringelstetter, Myles Fennell, Juliane Merl‐Pham, Anna Artati, Stefanie Strasser, Ralph Garippa, Michael Witting, Hans Zischka, Joel A. Schick, Stefanie M. Hauck, Michael P. Menden, Michelle Vincendeau, Brent R. Stockwell, Kamyar Hadian

**Affiliations:** ^1^ Cell Signaling and Chemical Biology, Research Unit Signaling and Translation Helmholtz Zentrum München Neuherberg Germany; ^2^ Institute of Virology Helmholtz Zentrum München Neuherberg Germany; ^3^ Computational Health Center Helmholtz Zentrum München Neuherberg Germany; ^4^ Department of Biological Sciences Columbia University New York New York USA; ^5^ Gene Editing & Screening Core Facility Memorial Sloan Kettering Cancer Center New York New York USA; ^6^ Metabolomics and Proteomics Core Helmholtz Zentrum München Neuherberg Germany; ^7^ Chair of Analytical Food Chemistry, TUM School of Life Sciences Technical University of Munich Freising‐Weihenstephan Germany; ^8^ Institute of Molecular Toxicology and Pharmacology Helmholtz Zentrum München Neuherberg Germany; ^9^ Institute of Toxicology and Environmental Hygiene School of Medicine and Health Technical University Munich Munich Germany; ^10^ Genetics and Cellular Engineering Group, Research Unit Signaling and Translation Helmholtz Zentrum Munich Neuherberg Germany; ^11^ Department of Biochemistry and Pharmacology Bio21 Molecular Science and Biotechnology Institute The University of Melbourne Parkville Victoria Australia; ^12^ Institute of Virology School of Medicine and Health Technical University Munich Germany; ^13^ Department of Chemistry Department of Pathology and Cell Biology Herbert Irving Comprehensive Cancer Center Irving Institute for Cancer Dynamics Data Science Institute Digestive and Liver Disease Research Center Columbia University New York New York USA

**Keywords:** ferroptosis, GPX4, lipid peroxidation, microRNA, miR‐940

## Abstract

Ferroptosis is a form of regulated cell death that is characterized by iron‐dependent lipid peroxidation. This process is regulated by specific metabolites, the lipid composition of the cells, redox‐active iron, and antioxidant mechanisms. Although numerous regulators have been identified over the past decade, exploring other mechanisms, particularly from non‐coding genomic regions, can build a thorough understanding of the multifaceted regulatory processes underlying ferroptosis. MicroRNAs (miRNAs) play a crucial role in gene regulation and cellular functions. Through a CRISPR KO screen, we identified miR‐940 as a negative regulator of ferroptosis. Overexpression of miR‐940 in several cell lines consistently suppressed ferroptosis induced by system x_c_^−^ inhibition. Notably, multiple cancer patient cohorts with elevated miR‐940 levels exhibit reduced survival. Integrated bioinformatic, transcriptomic, and proteomic analyses revealed that miR‐940 decreases the expression of ACSL4, LPCAT3, DMT1, and NCOA4, and simultaneously increases levels of GPX4. Pharmacological inhibition of GPX4 attenuated the protective effect of miR‐940, indicating that its primary anti‐ferroptotic activity is mediated through GPX4. Overall, these mechanistic insights link gene rewiring to reduced levels of redox‐active iron and diminished lipid peroxidation, mediating ferroptosis suppression. These findings provide a defined regulatory network, presenting a novel target for therapeutic exploration in susceptible cancers.

## Graphical abstract

A CRISPR‐based screening identified miR‐940 as a critical suppressor of ferroptosis in cancer. By coordinating the downregulation of pro‐ferroptotic genes with the upregulation of GPX4, miR‐940 establishes a regulatory network that protects against ferroptosis and correlates with poor clinical outcomes in distinct cancer entities. This study unveils a non‐coding mechanism for ferroptosis resistance, offering new avenues for targeted cancer therapy.

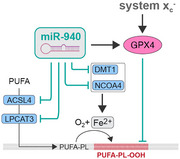

## 1 Introduction

Cell homeostasis is dependent on cell division and cell death. The past two decades have shown that besides apoptosis, several other cell death modalities are executed in a regulated fashion [1]. Ferroptosis is a regulated cell death pathway that is based on iron‐dependent lipid peroxidation [2, 3, 4]. It is initiated by the presence of phospholipids with polyunsaturated fatty acyl tails (PUFA‐PLs), redox‐active iron, and a defective lipid peroxide repair system [5]. PUFAs are incorporated into phospholipids by the action of the enzymes acyl‐CoA synthetase long‐chain family member 4 (ACSL4) and lysophosphatidylcholine acyltransferase 3 (LPCAT3) [6]. Iron is transported into cells by endocytosis mediated by transferrin receptor protein 1 (TfR1) and released from endosomes by divalent metal transporter 1 (DMT1) [4]. Iron is then stored in a non‐redox‐active form in iron‐ferritin complexes and released from ferritin in a process called ferritinophagy, mediated by nuclear receptor coactivator 4 (NCOA4) [6]. Interestingly, TfR1 has also been shown to act as a potent marker to detect ferroptosis [7, 8]. To prevent ferroptosis, cells have developed several ways to protect themselves from lipid peroxidation. The system x_c_^−^/GPX4/glutathione axis is the central ferroptosis inhibitory mechanism, where glutathione peroxidase 4 (GPX4) uses glutathione (GSH) to reduce lipid hydroperoxides to the corresponding alcohol forms [4]. In addition, there are at least two ferroptosis inhibitory modules that act independently of GPX4, namely the FSP1/ubiquinol/vitamin‐K axis [9, 10, 11], and the GCH1/DHFR/tetrahydrobiopterin axis [12, 13]. Several synthetic ferroptosis inducers (IKE, RSL3, ML162, and FINO_2_) as well as inhibitors (Fer‐1 and Lip‐1) have been developed that can modulate ferroptosis sensitivity [1]. Additionally, approved drugs have been repurposed to inhibit ferroptosis, such as seratrodast [14].

Several transcriptional factors control the expression of key ferroptosis regulators to balance ferroptosis induction. Examples are the nuclear factor‐erythroid 2‐related factor 2 (NRF2) [6], as well as nuclear receptors including the farnesoid X receptor (FXR) [15], the retinoic acid receptor (RAR) [16] or the estrogen receptor (ER) [17, 18]. Gene expression can also be regulated by microRNAs (miRNAs), which either degrade the mRNA or target the untranslated regions (UTRs) of mRNA transcripts to prevent their translation [19]. Recent years have seen growing interest in miRNA‐mediated regulation in many fields of research, including ferroptosis. Although several studies have identified specific miRNAs that influence the ability of cells to undergo ferroptosis [20], there is an opportunity to expand our knowledge of this class of regulators.

The microRNA miR‐940 is located on chromosome 16p13.3 and is known to exhibit dysregulated expression in various diseases, including many types of cancer [21]. It binds to the 3’ untranslated region (UTR) of target protein‐coding genes, long non‐coding RNAs, and circular RNAs, thereby affecting their transcription or post‐transcriptional regulation [21]. Unlike many existing protein regulators that control tumor progression, the expression of miR‐940 can be increased or decreased in cancer, depending on the tissue and cell type. Its expression is decreased in hepatocellular carcinoma [22] and non‐small‐cell lung cancer [23], but increased in gastric [24] and cervical cancer [25]. This opposing role of miR‐940 has also been described in tumor metastasis [26], cell cycle progression [25], the epithelial‐mesenchymal transition [27], among others. MiR‐940 affects signaling pathways by either directly targeting molecules such as MAPK1 [28], or indirectly by inhibiting CBL‐b and c‐CBL, resulting in PD‐L1 upregulation and an increase in STAT3 and AKT [29]. In addition to signal transduction, potential mechanisms by which miR‐940 regulates tumorigenesis include the induction of DNA damage [30], interference with folate metabolism through the downregulation of MTHFD2 [31], and the silencing of transcription factors such as ZNF24 [24]. However, a connection to ferroptosis regulation has never been reported.

In this study, we identified miR‐940 as a key modulator of ferroptosis by systematically integrating data from a CRISPR KO screen, transcriptomics, and proteomics. We demonstrate that miR‐940 upregulates GPX4, while concurrently reducing levels of ferroptosis‐promoting genes including ACSL4, LPCAT3, DMT1, and NCOA4. Together, this regulation by miR‐940 reduces ferroptosis. By defining this regulatory architecture, these findings position miR‐940 as a functionally defined regulatory hub controlling ferroptosis. This mechanistic insight provides a precise molecular rationale to therapeutically target lipid peroxidation pathways in susceptible cancers.

## 2 Results

### 2.1 CRISPR KO Screen Identifies miR‐940 as a Negative Regulator of Ferroptosis

**FIGURE 1:**
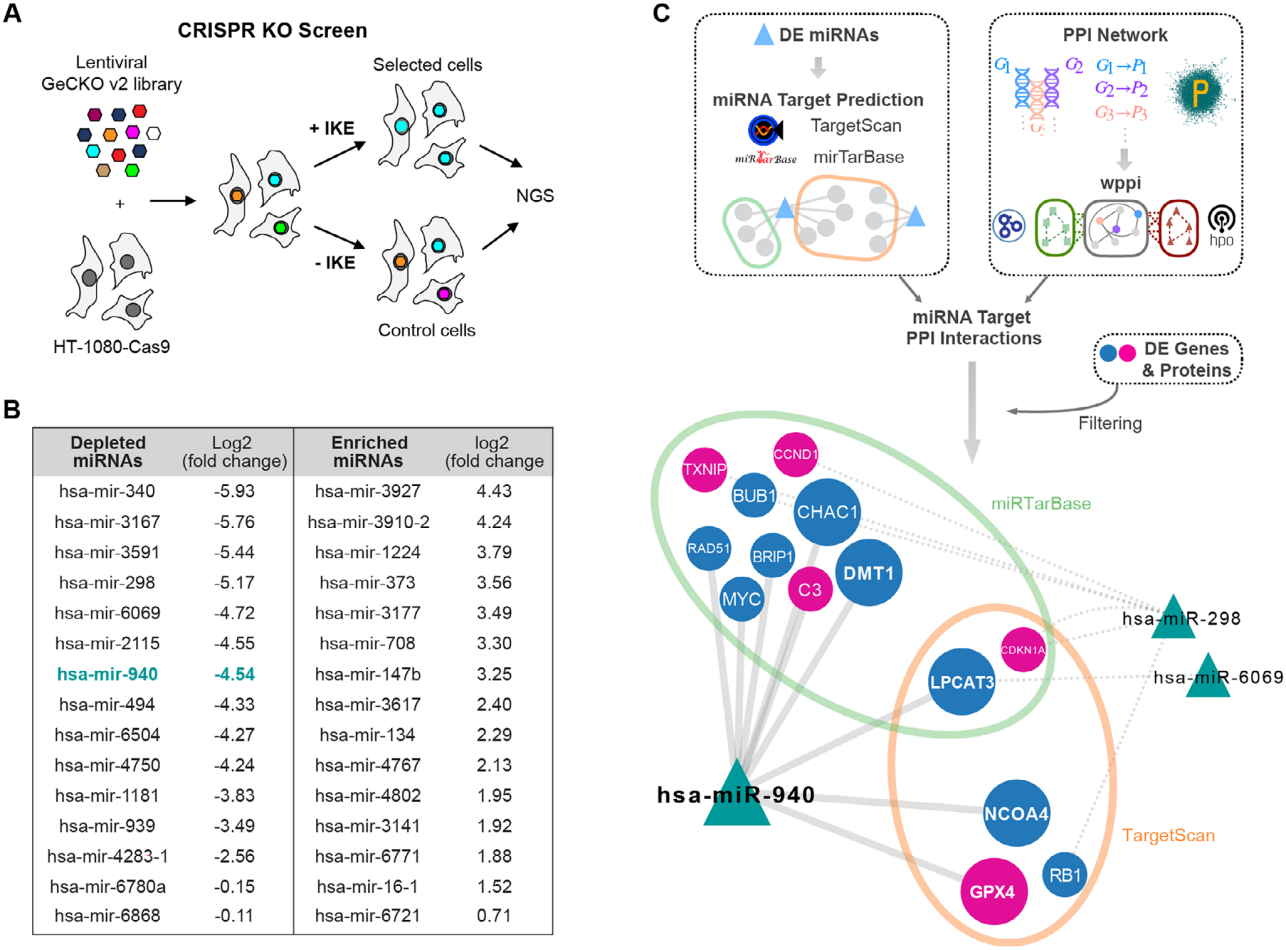
CRISPR KO screen identifies miR‐940 as a negative regulator of ferroptosis. (A) Illustration of the genome‐wide CRISPR knockout screen using the GeCKO v2 human CRISPR knockout pooled library. (B) Differentially regulated miRNAs resulting from the CRISPR knockout screen. (C) Illustration of the bioinformatic pipeline for miRNA target prediction and protein‐protein interaction (PPI) analysis. Gene targets of the differentially regulated miRNAs were retrieved from the TargetScan and miRTarBase databases. Validated gene targets from miRTarBase were leveraged in a weighted PPI (WPPI) network analysis to expand the candidate gene list. Focusing on targets among differentially expressed genes and proteins (FDR = 1% and |log2 fold‐change| > 1), a total of 14 genes were identified as targets of hsa‐miR‐940, hsa‐miR‐298, and hsa‐miR‐6069. In the network illustration, blue nodes indicate up‐regulated genes, red nodes indicate down‐regulated genes, and triangles represent miRNAs. Large nodes represent the 5 genes identified by miRTarBase and TargetScan (i.e., CHAC1, DMT1, LPCAT3, NCOA4, and GPX4), while small nodes represent the 9 new genes identified through the WPPI network analysis (i.e., TXNIP, CCND1, BUB1, RAD51, BRIP1, MYC, C3, CDKN1A, and RB1).

To discover novel ferroptosis regulators, we performed a genome‐wide CRISPR knockout (KO) screen in this study using the GeCKO v2 human CRISPR knockout pooled library [32]. Cas9‐expressing HT‐1080 cells, a widely used ferroptosis model, were transduced with the library and treated with the ferroptosis inducer Imidazole ketone erastin (IKE) [33] or DMSO. After 18 h, genomic DNA of surviving cells was extracted and subjected to next generation sequencing (NGS) (Figure 1A). Analysis of the abundance of gRNAs associated with miRNAs revealed a set of 30 differentially expressed gRNAs upon ferroptosis induction. Here, 15 of these were significantly enriched, suggesting that the corresponding miRNAs are pro‐ferroptotic, and the other 15 were significantly depleted, indicating that the corresponding miRNAs are negative regulators of ferroptosis (Figure 1B).

To predict the most promising miRNA candidates that may be involved in ferroptosis‐related pathways, we performed an in‐depth bioinformatics analysis. We seeded 95 genes/proteins with a known contribution in ferroptosis regulation into a functional network analysis (Figure 1C). We then performed miRNA target prediction and protein‐protein interaction (PPI) analysis, ranking the candidate genes based on their association score with the ferroptosis‐related seed genes. These analyses revealed that miR‐940 showed the strongest association with ferroptosis regulatory proteins including DMT1, LPCAT3, NCOA4, and GPX4 (Figure 1C). This prompted us to select this miRNA for further analysis.

### 2.2 MiR‐940 Inhibits IKE‐Induced Ferroptosis

**FIGURE 2:**
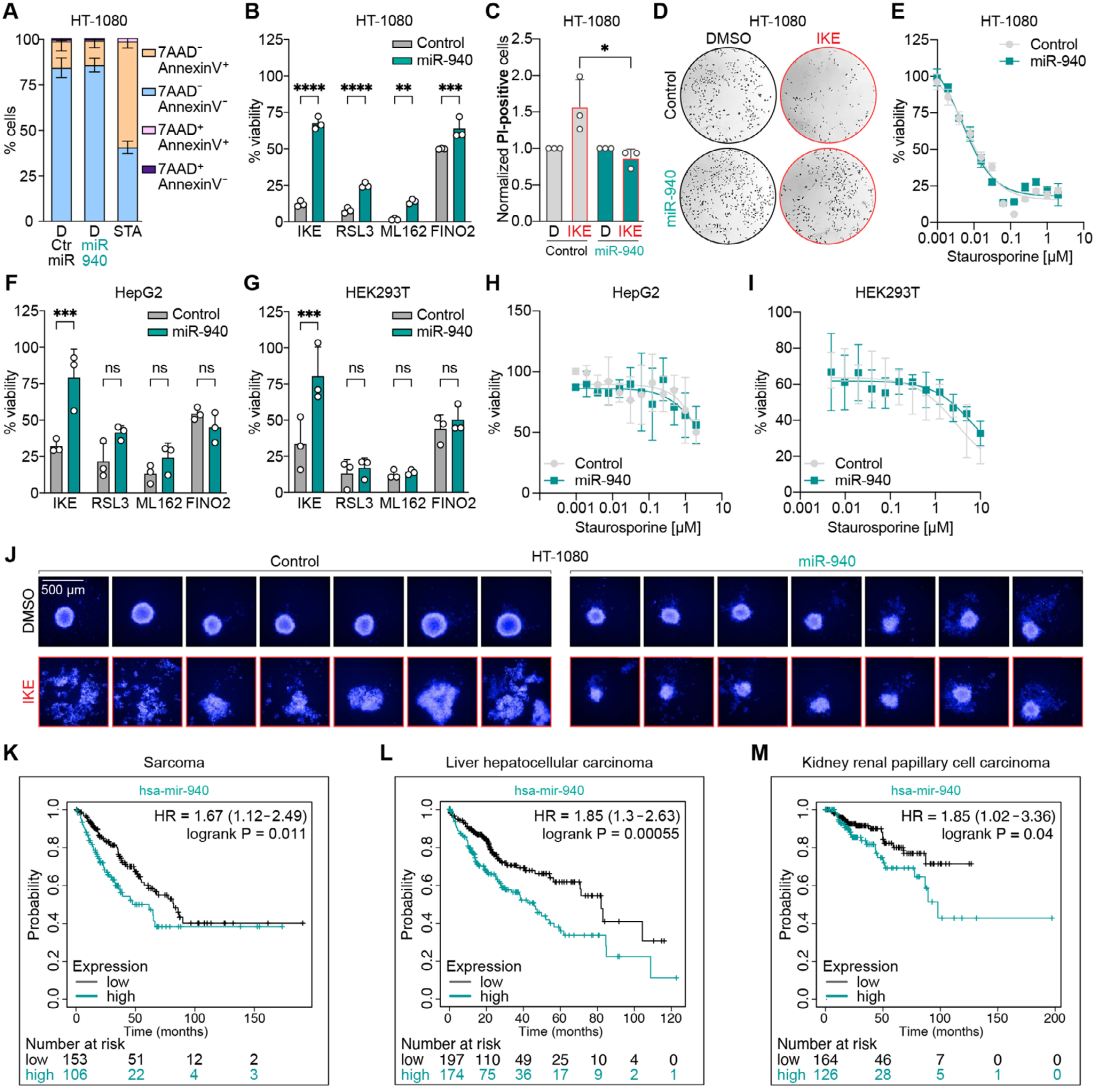
miR‐940 inhibits IKE‐induced ferroptosis (A) AnnexinV/7‐AAD staining in pre‐miR‐940‐ and control‐transfected HT‐1080 cells 24 h post transfection. AnnexinV positive control was included by the addition of staurosporine (STA, 1 µM), data are mean ± SD of n = 3 biological replicates. (B) Inhibition of ferroptosis in pre‐miR‐940‐ and control‐transfected HT‐1080 cells treated with 0.3 µM IKE, 0.25 µM RSL3, 0.25 µM ML162 and 0.6 µM FINO_2_; ^****^ = *p* ≤ 0.0001, ^***^ = *p* ≤ 0.001, ^**^ = *p* ≤ 0.01, 2way ANOVA, data are mean ± SD of n = 3 biological replicates. (C) Measurement of PI‐positive cells upon induction of ferroptosis (2 µM IKE) in pre‐miR‐940‐ and control‐transfected HT‐1080 cells, ^*^ = *p* ≤ 0.05, 2way ANOVA, data are mean ± SD of n = 3 biological replicates. (D) Crystal violet staining of pre‐miR‐940‐ and control‐transfected HT‐1080 cells after treatment with 2 µM IKE for 24 h. (E) Dose dependent induction of apoptosis with staurosporine in pre‐miR‐940‐ and control‐transfected HT‐1080 cells. (F,G) Inhibition of ferroptosis in pre‐miR‐940‐ and control‐transfected HepG2 (F) and HEK293T (G) cells treated with (F) 2.5 µM IKE, 0.5 µM RSL3, 0.25 µM ML162, and 2.5 µM FINO_2_ and (G) 0.6 µM IKE, 0.25 µM RSL3, 0.25 µM ML162, and 1.25 µM FINO_2_; ^***^ = *p* ≤ 0.001, ns = *p* > 0.05, 2way ANOVA, data are mean ± SD of n = 3 biological replicates. (H,I) Dose dependent induction of apoptosis with staurosporine in pre‐miR‐940‐ and control‐transfected HepG2 (H) and HEK293T (I) cells. (J) 3D spheroid formation of pre‐miR‐940‐ and control‐transfected HT‐1080 cells treated with 2 µM IKE, median intensity of Hoechst signal visualized. (K,L,M) Query of Kaplan‐Meier (KM) plotter to analyze the correlation of miR‐940 expression and patient survival in sarcoma (K), liver hepatocellular carcinoma (L), and kidney renal papillary cell carcinoma (M). Cut‐points for high versus low levels were determined using the median expression of hsa‐mir‐940. No restrictions applied (stage all; gender all; race all; grade all; mutation burden all). All cell types included.

Previous studies suggested that miR‐940 may induce apoptosis [34, 35]. In contrast, using Annexin V/7‐AAD staining, we show that miR‐940 does not induce apoptosis in our experimental setting (Figure 2A). Next, we performed a series of experiments to confirm a negative regulatory role of miR‐940 in ferroptosis. We transfected HT‐1080, HepG2 cells, and HEK293T cells with the precursor miRNA of miR‐940 (pre‐miR‐940) and detected a robust upregulation of miR‐940 transcripts (Supplementary Figure S1A). We used three different cell lines from three different tissue origins to demonstrate the generalizability of ferroptosis suppression by miR‐940. We then induced ferroptosis using IKE, RSL3, ML162 or FINO_2_. These molecules all selectively induced ferroptosis, because the specific ferroptosis inhibitor Fer‐1 fully restored cell viability when co‐treated with them (Supplementary Figure S1B). As expected from the screening results, ferroptosis induction by IKE was significantly rescued by miR‐940 overexpression in HT‐1080 cells (Figure 2B). Interestingly, ferroptosis induced by GPX4 inhibition (RSL3, ML162 or FINO_2_) was only slightly inhibited by miR‐940 (Figure 2B). IKE‐induced ferroptosis inhibition by miR‐940 was additionally demonstrated through PI staining (Figure 2C) and crystal violet staining (Figure 2D). Importantly, staurosporine‐induced apoptosis was not inhibited by miR‐940 in HT‐1080 cells (Figure 2E), suggesting specificity toward ferroptosis over apoptosis. These data could be confirmed using Annexin V/7‐AAD staining (Supplementary Figure S1C). To determine if this effect was cell line‐specific, we examined ferroptosis susceptibility after miR‐940 overexpression in two additional cell lines. HepG2 cells were also rescued by miR‐940 from IKE‐induced ferroptosis, but not after RSL3, ML162, and FINO_2_ treatment (Figure 2F). Similar results were obtained in HEK293T cells, namely, suppression of ferroptosis by miR‐940 upon IKE‐induced system x_c_^−^ inhibition only (Figure 2G). In both cell lines, HepG2 and HEK293T cells, miR‐940 had no effect on apoptosis (Figure 2H,I).

We next tested the inhibitory effect of miR‐940 on ferroptosis in a more physiological setting. Therefore, we used 3D spheroids of HT‐1080 cells, either transfected with pre‐miR‐940 or the control (seven spheroids per condition are depicted in Figure 2J). Spheroid formation in cells treated with IKE was impaired compared to the DMSO control. This effect could not be observed in cells transfected with pre‐miR‐940, which showed no difference in the spheroid integrity and size between DMSO and IKE treatment (Figure 2J). Notably, more cells migrated out of the spheroids when transfected with pre‐miR‐940, which is likely the result of altered pathways by miR‐940, thereby facilitating cell migration [36]. Hence, we validated the anti‐ferroptotic effect of miR‐940 also in a three‐dimensional setup that more closely reflects physiological conditions. We also queried the KM Plotter [37, 38, 39] and analyzed the correlation between miR‐940 expression and patient survival. Patients with higher levels had worse survival rates in sarcoma (Figure 2K), liver hepatocellular carcinoma (Figure 2L), and kidney renal papillary cell carcinoma (Figure 2M). These patient data align well with our HT‐1080 (fibrosarcoma), HepG2 cells (liver hepatocellular carcinoma), and HEK293T (kidney cells), which exhibit ferroptosis suppression by miR‐940.

#### 2.2.1 MiR‐940 Modulates Key Regulators of Ferroptotic Cell Death

**FIGURE 3:**
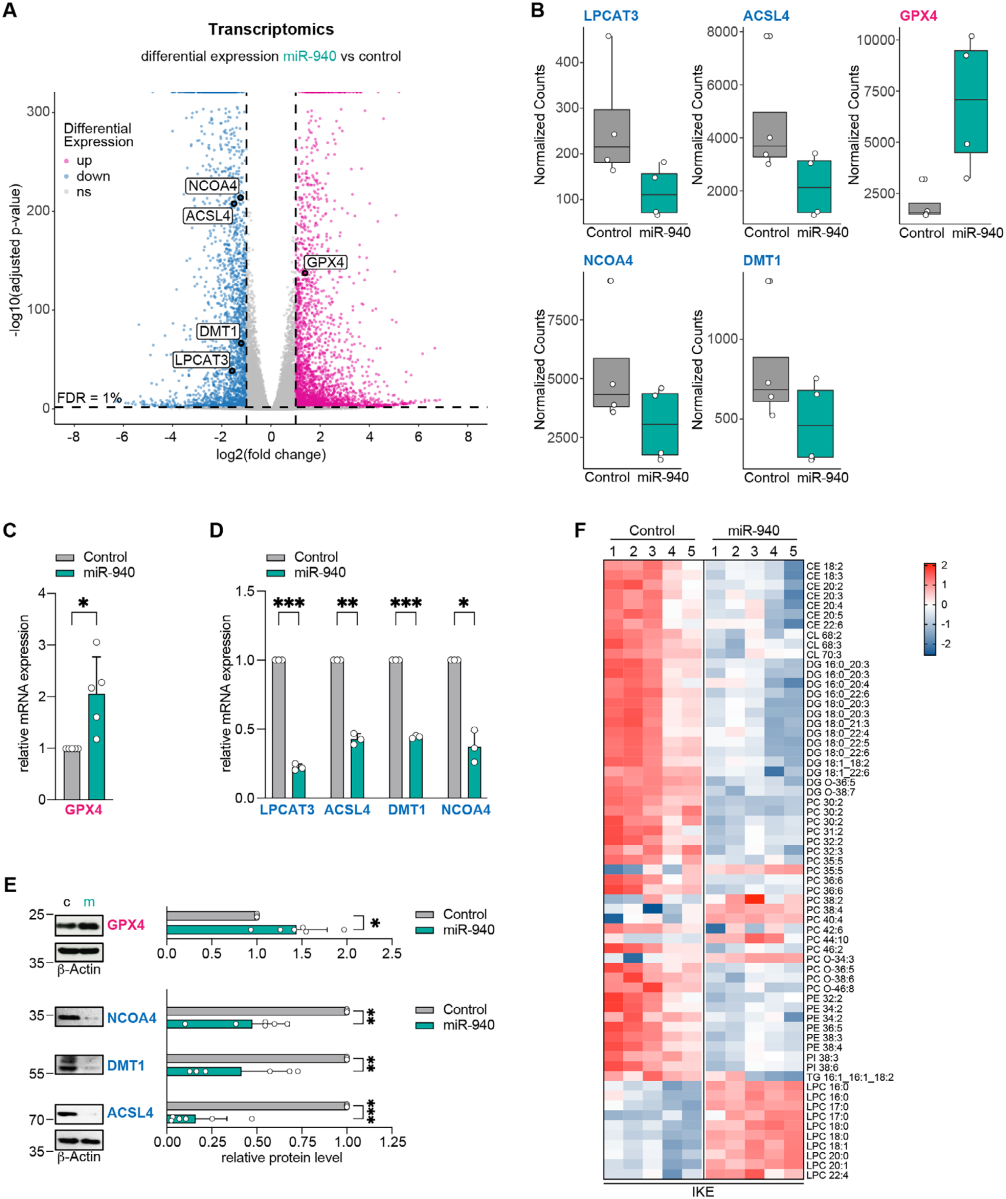
miR‐940 modulates key regulators of ferroptotic cell death. (A) Transcriptomics analysis of miR‐940‐overexpressing HT‐1080 cells show downregulation of NCOA4, ACSL4, DMT1, and LPCAT3 and upregulation of GPX4, significance at False Discovery Rate (FDR) of 1% and absolute value of log2 fold‐change (log2FC) > 1, Wald test adjusted with Benjamini‐Hochberg correction. (B) Normalized counts of transcriptomics analysis of LPCAT3, ACSL4, DMT1, NCOA4, and GPX4, data are mean ± SD of n = 4 biological replicates. (C) qRT‐PCR results of GPX4 in miR‐940 transfected cells, ^*^ = *p* ≤ 0.05, unpaired t‐test with Welch's correction, data are mean ± SD of n = 3 biological replicates. (C) qRT‐PCR results of GPX4 in miR‐940 transfected cells, ^*^ = *p* ≤ 0.05, unpaired t‐test with Welch's correction, data are mean ± SD of n = 5 biological replicates. (D) qRT‐PCR results of LPCAT3, ACSL4, DMT1, and NCOA4 in miR‐940‐overexpressing cells, ^*^ = *p* ≤ 0.05, ^**^ = *p* ≤ 0.01, ^***^ = *p* ≤ 0.001, unpaired t‐test with Welch correction, data are mean ± SD of n = 3 biological replicates. (E) Western Blot analysis of miR‐940‐overexpressing HT‐1080 cells with staining against GPX4, NCOA4, DMT1, and ACSL4 relative to β‐Actin levels, ^*^ = *p* ≤ 0.05, ^**^ = *p* ≤ 0.01, ^***^ = *p* ≤ 0.001, unpaired t‐test with Welch correction, data are mean ± SD of n = 6 biological replicates. Full western blots can be found in the supplementary (Supplementary Figure S6). (F) Lipidomics analyses of pre‐miR‐940‐ and control‐transfected HT‐1080 cells treated with 2 µM IKE show depletion of ferroptosis‐relevant PUFA‐containing phospholipids upon miR‐940 overexpression, CE cholesteryl ester, CL cardiolipin, DG diacylglyceride, PC phosphatidylcholine, PE phosphatidylethanolamine, PI phosphatidylinositol, TG triglyceride, LPC lysophosphatidylcholin, depicted are Z‐scores with *p* ≤ 0.05, two‐tailed t‐test, n = 5 biological replicates.

To further investigate the role of miR‐940 in ferroptosis‐related pathways, we subjected total RNA from pre‐miR‐940‐ and control‐transfected cells to RNA sequencing (RNAseq). Transcriptomics analysis revealed the downregulation of four ferroptosis‐promoting target genes: LPCAT3, DMT1, NCOA4, and ACSL4 (Figure 3A). This largely aligned with the prediction of our bioinformatics pipeline (Figure 1C). Transcriptomics also showed that GPX4, the central gatekeeper of ferroptosis, was upregulated upon pre‐miR‐940 overexpression (Figure 3A), which aligns with the bioinformatic prediction. Normalized transcript counts from the RNAseq data are shown in Figure 3B. We did not assess direct 3' UTR binding for these transcripts; thus, direct miR‐940‐mRNA interactions remain to be established. However, predicted miR‐940 binding sites were identified in the 3′ UTRs of the ferroptosis regulators *NCOA4*, *ACSL4*, *LPCAT3*, *DMT1*, and *GPX4* (Supplementary Figure S2 and Supplementary Table S1). Notably, *NCOA4* revealed a high‐confidence 8mer site predicted by both TargetScan and miRDB, while *DMT1* was previously validated as a direct miR‐940 target [40]. Along with transcriptomics, we performed proteomics and observed downregulation of ACSL4 protein levels. Unfortunately, the other proteins were not detected due to limited resolution (Supplementary Figure S3).

To confirm the omics data, the mRNA levels of all differentially expressed target regulators were analyzed by qRT‐PCR. Significant upregulation of GPX4 (Figure 3C) and downregulation of LPCAT3, ACSL4, DMT1, and NCOA4 (Figure 3D) could be confirmed upon transfection with pre‐miR‐940. Similar results were obtained in HepG2 cells and HEK293T cells (Supplementary Figure S4A,B). Finally, we investigated whether miR‐940 reduces the protein levels of the aforementioned target proteins by performing western blot analysis and quantification. Overexpression of miR‐940 significantly increased GPX4 protein levels, protein levels of NCOA4 spliced variant, DMT1, and ACSL4 were significantly decreased (Figure 3E). Full western blots can be found in the supplementary (Supplementary Figure S6). These findings demonstrate that miR‐940 modulates key regulators of ferroptotic cell death, as detected on RNA as well as protein levels. Interestingly, lipidomics analyses of control‐miRNA‐ versus miR‐940‐transfected cells demonstrate that several ferroptosis‐relevant PUFA‐containing phospholipids are depleted upon miR‐940 overexpression (Figure 3F). According to our data, this largely correlates with a reduction in ACSL4 and LPCAT3 levels. Yet, reflecting on the data that miR‐940 largely failed to rescue from ferroptosis upon GPX4 inhibition, this fact sets GPX4 as the major mechanism by which miR‐940 suppresses ferroptosis, because upregulated GPX4 proteins (Figure 3E) are still inhibited by RSL3, ML162 and FINO_2_.

### 2.3 MiR‐940 Suppresses Iron Accumulation and Lipid Peroxidation

**FIGURE 4:**
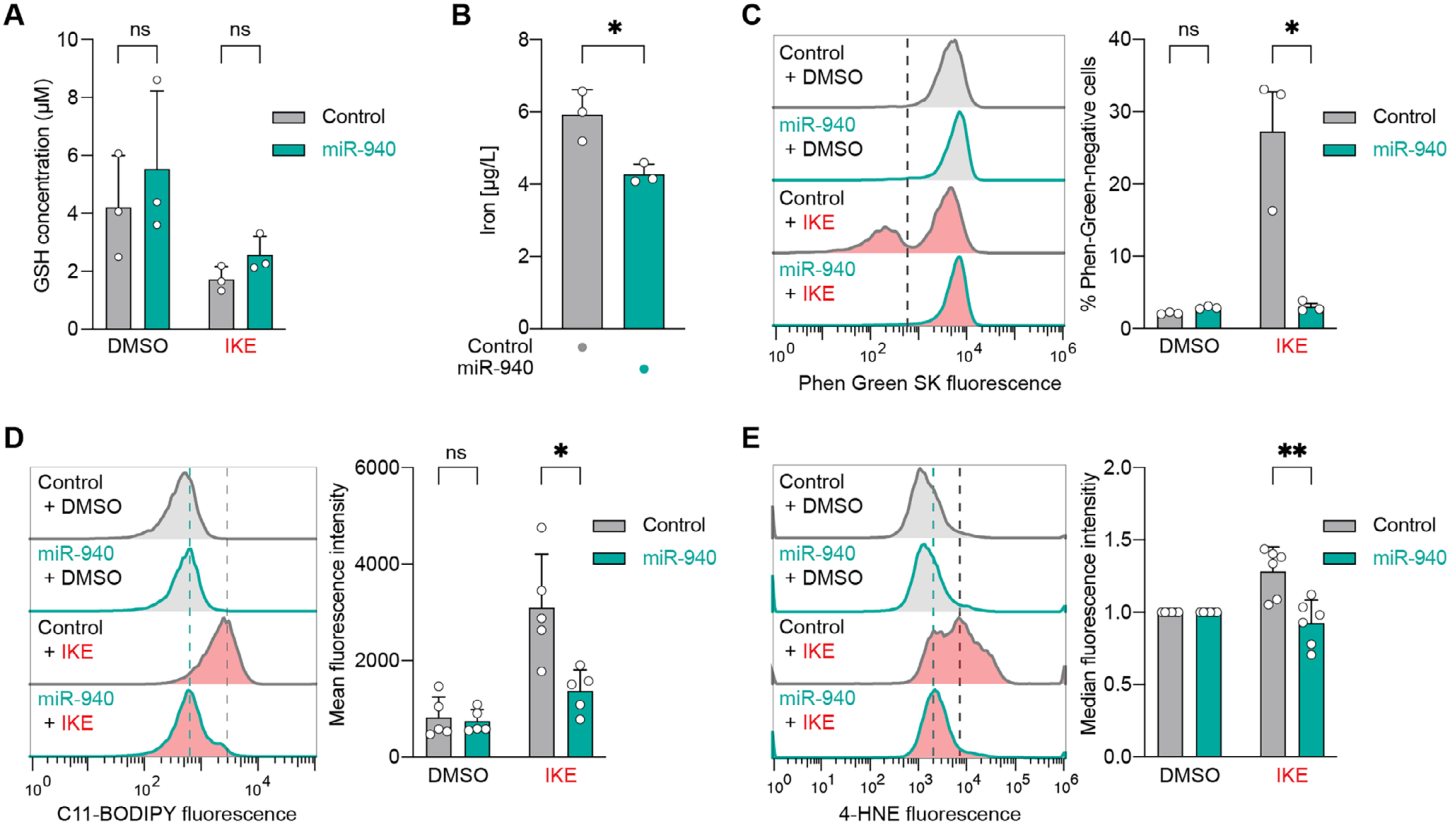
miR‐940 suppresses iron accumulation and lipid peroxidation (A) Measurements of GSH concentration upon treatment with 2 µM IKE in both control and pre‐miR‐940 transfected H1080 cells, ns = p > 0.05, 2way ANOVA, data are mean ± SD of n = 3 biological replicates. (B) Measurements of iron concentration via ICP‐OES show reduced levels in miR‐940‐overexpressing cells compared to control cells, ^*^ = *p* ≤ 0.05, unpaired t‐test with Welch's correction, data are mean ± SD of n = 3 biological replicates. (C) Analysis of iron levels via the heavy metal indicator PhenGreen SK Diacetate in pre‐miR‐940 transfected cells after treatment with 2 µM IKE compared to control cells, ns = *p* > 0.05, ^*^ = *p* ≤ 0.05, 2way ANOVA, data are mean ± SD of n = 3 biological replicates. (D) C11‐BODIPY sensor of lipid peroxidation upon treatment with 2 µM IKE in miR‐940 overexpressing cells, ns = *p* > 0.05, ^*^ = *p* ≤ 0.05, 2way ANOVA, data are mean ± SD of n = 5 biological replicates. (E) Detection of lipid peroxidation via antibody for 4‐HNE in miR‐940 overexpressing cells upon treatment with 2 µM IKE, ^**^ = *p* ≤ 0.01, 2way ANOVA, data are mean ± SD of n = 6 biological replicates.

To assess the mechanism behind the anti‐ferroptotic actions of miR‐940, we first investigated glutathione levels within HT‐1080 cells following treatment with IKE. No significant increase in GSH concentration was observed in miR‐940‐transfected versus control cells. Both, miR‐940‐transfected cells and control cells, showed decreased GSH levels upon IKE‐induced ferroptosis, indicating that miR‐940 has no direct influence on GSH levels (Figure 4A).

Next, iron levels of HT‐1080 cells transfected with miR‐940 were measured via ‘inductively coupled plasma optical emission spectroscopy’ (ICP‐OES), as well as using the heavy metal indicator PhenGreen SK Diacetate. Both assays revealed a significant decrease in iron levels in cells overexpressing miR‐940 before (Figure 4B) and after ferroptosis induction (Figure 4C). Moreover, lipid peroxidation was detected via staining with the C11‐BODIPY sensor and a 4‐HNE immunostaining after inducing ferroptotic cell death with IKE in HT‐1080 cells. Both assays showed an induction of lipid peroxidation in IKE‐treated control cells. Importantly, overexpression of miR‐940 significantly suppressed levels of lipid peroxidation as detected by both techniques (Figure 4D,E). We also assessed lipid peroxidation using the C11‐BODIPY sensor in HepG2 cells. These cells are more resistant to ferroptosis, which results in a smaller fluorescence shift. Nevertheless, a significant reduction of IKE‐induced lipid peroxidation was detected in miR‐940‐ overexpressing cells (Supplementary Figure S5A). Interestingly, similar to viability measurement (Figure 2B,F), miR‐940 was not able to reduce lipid peroxidation induced by GPX4 inhibition (RSL3) in HT‐1080 and HepG2 cells (Supplementary Figure S5B).

**FIGURE 5:**
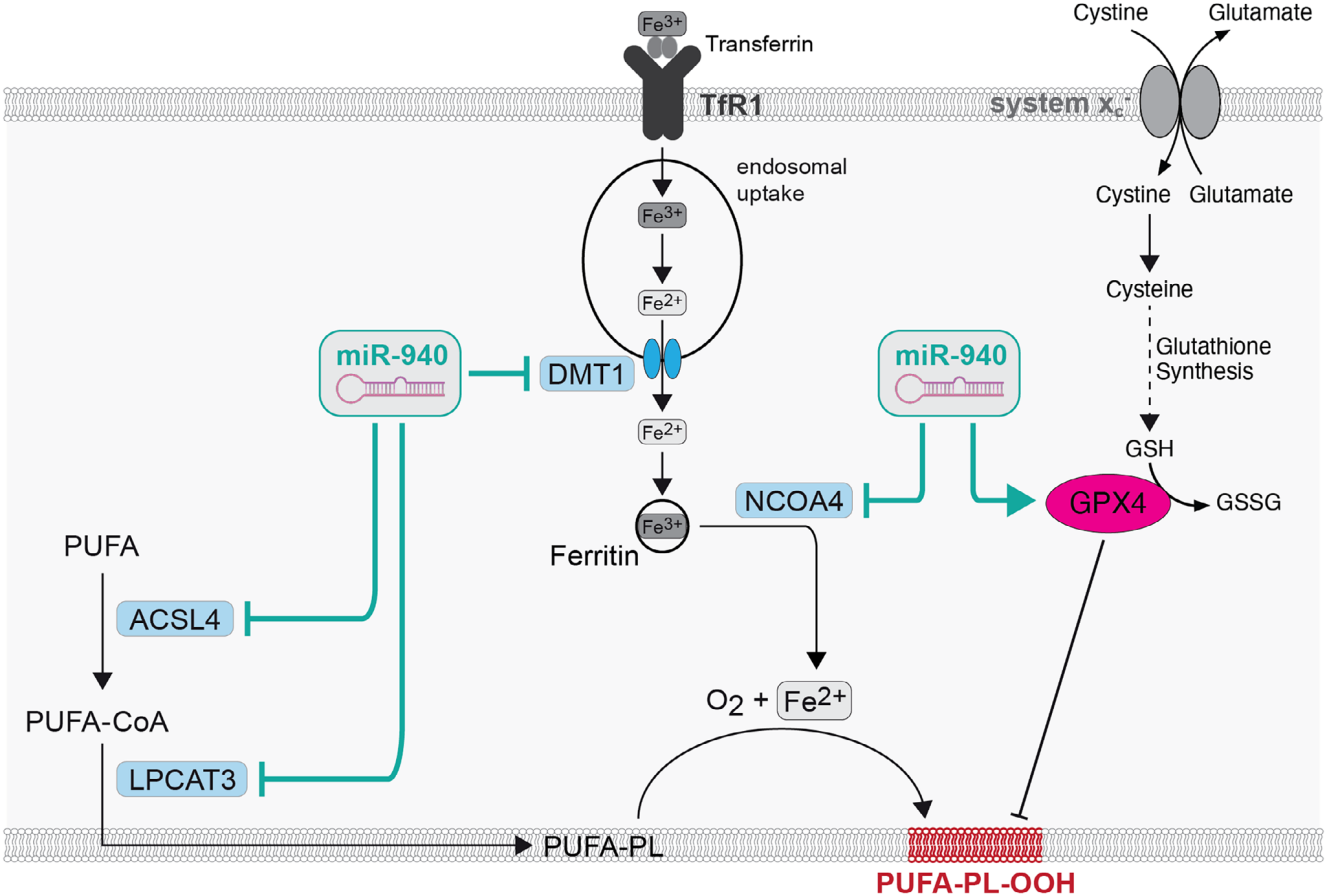
Working model of how miR‐940 suppresses ferroptosis.

Together, these findings suggest that miR‐940 inhibits ferroptotic cell death through three mechanisms: 1) enhanced repair of PLOOH due to upregulation of GPX4, 2) decreased levels of redox‐active iron associated with reduced expression of NCOA4 and DMT1, and 3) a reduction in PUFA‐PL levels through downregulation of LPCAT3 and ACSL4 (Figure 5).

## 3 Discussion

Our data suggest that miR‐940 is a previously unrecognized cellular inhibitor of ferroptosis (Figure 2), acting through the coordinated modulation of the gene expression of ferroptosis regulators, especially the upregulation of the central gatekeeper of ferroptosis, namely GPX4. Rather than affecting individual effectors, it appears that miR‐940 influences a broader regulatory architecture, the major hallmarks of ferroptosis, which controls cellular susceptibility to oxidative cell death. Therefore, we propose that miR‐940 is a potent, novel cellular suppressor of ferroptotic cell death (Figure 5).

This illustration shows how miR‐940 suppresses ferroptosis by downregulating ACSL4 and LPCAT3, which leads to reduced PUFA‐PL levels in the cell membrane. It also inhibits DMT1 to limit iron uptake, and NCOA4 to reduce ferritin degradation and thus the release of the labile, redox‐active iron pool. This results in lower Fe^2^^+^ levels. Finally, it upregulates GPX4, which reduces lipid peroxides and counteracts ferroptosis.

Through integrative analysis, we mechanistically unraveled that miR‐940 alters the expression profiles of several regulators involved in phospholipid processing and iron handling, both of which essentially govern vulnerability to lipid peroxidation (Figure 3). This suggests that small non‐coding RNAs can have wide‐ranging effects on cell fate by simultaneously targeting functionally linked molecular processes. Notably, ACSL4 and LPCAT3 are pivotal enzymes in determining phospholipid composition and susceptibility to peroxidation [3, 41], while DMT1 and NCOA4 influence intracellular iron availability [4], two critical nodes in ferroptotic sensitivity. The simultaneous repression of these targets by miR‐940 suggests the existence of a robust evolutionary mechanism to avoid lipid peroxidation driven cell death. While the exact biophysical binding dynamics were not assessed via reporter assays, the functional multi‐omic regulation of this network is clear. Especially, the reduction of ACSL4 and LPCAT3 by miR‐940 has the consequence that ferroptosis‐relevant PUFA‐containing phospholipids are largely depleted upon miR‐940 overexpression as evident from lipidomics analysis (Figure 3F). Furthermore, the simultaneous upregulation of the selenoenzyme GPX4 by miR‐940 (Figure 3) is interesting, given its central role in ferroptosis inhibition [1, 3, 4]. Since miR‐940 largely failed to protect cells from ferroptosis after GPX4 inhibition (viability and lipid peroxidation), these results suggest that GPX4 is the main axis through which miR‐940 suppresses ferroptosis. The elevated levels of GPX4 protein upon miR‐940 overexpression will still be susceptible to inhibition by RSL3, ML162, and FINO_2_. However, the exact mechanism by which miR‐940 increases GPX4 expression remains unclear, it suggests the existence of indirect regulatory networks or the possibility of miR‐940‐mediated suppression of cellular GPX4 inhibitors; for example, an E3 ligase that triggers degradation of GPX4 by the proteasome. In some cases, miRNAs may also increase protein levels, which would be a direct effect. Thus, future investigation into upstream regulators and feedback loops involving miR‐940 and GPX4 will help to decipher this process.

While this study provides mechanistic insight into the regulation of ferroptosis by miR‐940, some limitations should be acknowledged. Our findings were confirmed in three independent cell lines, largely relying on in vitro experimental systems. To provide pathological context, we show that increased miR‐940 expression correlates with decreased overall survival in several cancer patient cohorts, supporting the notion that inhibition of ferroptosis may negatively influence patient outcomes. Although direct in vivo validation should be conducted in future studies using animal models or patient‐derived systems to evaluate the systemic (patho)physiological significance of the regulation of ferroptosis by miR‐940, this study establishes the complex regulatory architecture that is a critical prerequisite. By detailing precisely how miR‐940 rewires the expression of GPX4 alongside essential iron and lipid regulators (ACSL4, LPCAT3, DMT1, and NCOA4), we provide a validated molecular rationale for modulating ferroptosis. This mechanistic foundation ensures that future in vivo designs including miR‐940 to target lipid peroxidation pathways in susceptible cancers, are mechanistically driven rather than purely empirical. Furthermore, while our lipidomics data demonstrate a depletion of PUFA‐phospholipids and pharmacological assays indicate a primary anti‐ferroptotic role for GPX4, the exact contribution of ACSL4‐mediated lipid remodeling remains to be fully defined. Unambiguously decoupling the specific effects of ACSL4‐driven lipid remodeling from GPX4 upregulation remains an important area for future investigation. Lastly, although our data identify key factors associated with ferroptosis downstream of miR‐940 and demonstrate that miR‐940 does not influence apoptosis under our experimental conditions, potential off‐target effects or additional roles of miR‐940 in other cell death pathways cannot be completely ruled out.

From a translational perspective, miR‐940 may represent a promising target in cancer entities such as sarcoma, liver hepatocellular carcinoma or kidney cancer (Figure 2K–M), where resistance to ferroptosis contributes to therapeutic failure. Inhibiting miR‐940 may increase tumor sensitivity to ferroptosis inducers. In line with this, the elevated expression of miR‐940 could serve as a predictive biomarker for identifying patients who are likely to respond to miR‐940‐modulating therapies in combination with ferroptosis‐based medicines [42]. Combining miR‐940 antagonism with ferroptosis‐inducing regimens could increase anti‐tumor efficacy and open new ways of overcoming drug resistance. These concepts provide a framework for future preclinical and clinical oncology studies exploring miR‐940‐targeted strategies.

In summary, this study identifies miR‐940 as a potent regulator that limits ferroptotic cell death by reshaping lipid and iron homeostasis. These findings broaden our understanding of the role of microRNAs in controlling redox‐sensitive death pathways and emphasize the therapeutic potential of targeting miR‐940 in diseases associated with ferroptosis. Future efforts should focus on defining the upstream signals that govern miR‐940 expression and analyzing its interactions with other non‐coding RNAs and transcription factors. A better understanding of this regulatory landscape will provide a deeper insight into the biology of ferroptosis and could reveal new opportunities for precision therapies in cancer.

## 4 Experimental Section

### 4.1 Cell Culture

HT‐1080 cells (RRID:CVCL_0317), HepG2 cells (RRID:CVCL_0027), and HEK293T cells (RRID:CVCL_0063) were purchased from ATCC and grown in Dulbecco's Modified Eagle's medium (Thermo Fisher Scientific) containing 10% fetal bovine serum (Gibco), 1% Penicillin‐Streptomycin (Thermo Fisher Scientific), and supplemented with 1% non‐essential amino acids (Sigma) at 37°C with 5% CO_2_. Cells were regularly tested (including mycoplasma) and were completely contamination‐free.

### 4.2 Transfection of HT‐1080 Cells

If not indicated otherwise, 200 000 cells per well were seeded in 6‐well plates the day before transfection. Cells were transfected with 30 nM hsa‐miR‐940 pre‐miR or pre‐miR negative control #1 (AM17100, Ambion, Invitrogen) in 1 mL medium. Per well, 3 µL of 10 µM miRNA or negative control was diluted in 50 µL Opti‐MEM, and 50 µL 6% Lipofectamine RNAiMAX reagent in Opti‐MEM I reduced serum medium (all from Thermo Fisher Scientific) was added. The mix was incubated for 10 min at room temperature before dropwise addition to the cells. After another 5 min of incubation at room temperature, the plate was kept at 37°C. After 6 h, cells were washed with PBS, and fresh medium was added.

### 4.3 Cell Viability Assays

Transfected cells were harvested 48 h after transfection with 0.05% Trypsin‐EDTA and seeded into 384‐well plates at a density of 750 cells per well. After incubation for 6 h, different concentrations of ferroptosis inducers (IKE (Cayman Chemical), RSL3 (Sigma Aldrich), ML162 (Sigma Aldrich), FinO_2_ (Caymen Chemical)) or the apoptosis inducer staurosporine (Sigma Aldrich) were added to the cells. DMSO was used as a negative control. After 18 h at 37°C, CellTiter‐Glo 2.0 Reagent (Promega) was added directly into each well, and viability was assessed by luminescence measurement in an EnVision 2104 Multilabel plate reader (PerkinElmer).

### 4.4 AnnexinV + 7AAD Detection Assay

HT‐1080 cells were seeded in 6‐well plates at a density of 100,000 cells per well one day prior to miRNA transfection, with transfections performed as previously described. In +STA conditions, 20 h after transfection, 1 µM staurosporine was added to the cultures for 4 h. 24 h after transfection, media was removed from all wells before being washed with 1x PBS and then incubated in 1 mL Accutase (Gibco) for 5 min at 37^°^C. Lifted cells were then collected in media and centrifuged at 300 x g for 3 min, washed with 1x PBS, and again centrifuged at 300 x g for 3 min. AnnexinV assays were performed using the FITC Annexin V Apoptosis Detection Kit with 7‐AAD (Biolegend, Cat # 640922), according to the kit protocol. Cell pellets were resuspended in 100 µL AnnexinV binding buffer along with 5 µL of AnnexinV and 5 µL of 7AAD, before being briefly vortexed and incubated in the dark at room temperature for 15 min. 400 µL of AnnexinV binding buffer was subsequently added to each tube, and samples were analyzed using the CytoFLEX S flow cytometer (Beckman Coulter), using the 488 nm laser with 525/40 bandpass filter to detect AnnexinV‐FITC and the 561 nm laser with 610/20 bandpass filter to detect 7AAD. Data was analyzed using FlowJo (v10.10.0, BD Biosciences).

### 4.5 Crystal Violet Staining

HT‐1080 cells were seeded into a 6‐well plate at a density of 200,000 cells per well and transfected as described. After incubation for 24 h, cells were treated with 2 µM IKE. DMSO was used as a control treatment. After 6 h, the medium was removed, cells were washed with MilliQ water, and 1.5 mL crystal violet staining solution (0.5% in 20% methanol) was added. Cells were incubated at room temperature for 20 min and rinsed with MilliQ water until no residual crystal violet solution was visible. Images were taken with an EVOS FL fluorescence microscope.

### 4.6 Spheroid Formation

Transfected HT‐1080 cells were harvested 48 h after transfection, and 3 000 cells per well were seeded into 96‐well Round Bottom Ultra Low Attachment Microplates (Corning costar 7007). After 1 h of incubation at 37°C, cells were treated with 2 µM IKE or DMSO as control. 48 h after treatment nuclei were stained with Hoechst 33342 (Sigma Aldrich) at a dilution of 1:10,000 followed by 1 h incubation at 37°C. Spheroids were imaged using the Operetta High Content Screening System (Perkin Elmer) at 10x magnification. Image analysis was performed with Columbus Software (Perkin Elmer).

### 4.7 qRT‐PCR

Total RNA was isolated from transfected cells 48 h after transfection using InVitrap Spin Universal RNA Mini kit (Stratec Scientific) according to the manufacturer's instructions. Genomic DNA was removed by digestion with dsDNase (Promega) before reverse transcription of mRNA and miRNA. Maxima H Minus First Strand cDNA Synthesis Kit (Thermo Fisher Scientific) was used for the synthesis of first‐strand cDNA with oligo (dT)_18_ and random hexamer primers. Reverse transcription of miRNA was performed using MystiCq microRNA cDNA Synthesis Mix according to the manufacturer's instructions. Polyadenylation of microRNA was performed with the Poly(A) Polymerase (Thermo Fisher Scientific, AM2030). Subsequently, RNA was eliminated by digestion with RNase H for 30 min at 37C°. qRT‐PCR was performed on a LightCycler480 (Roche) with PowerUp SYBR Green Master Mix (Thermo Fisher Scientific) for cDNA transcribed from mRNA and SYBR Green Master Mix (Roche) for cDNA transcribed from miRNA. The primers used are listed in Supplementary Table S2. Expression levels of target genes were normalized to RNA Polymerase II, and quantification was carried out using the delta delta Cp method. For expression levels of miRNA, SNORD44 was used for normalization, respectively.

### 4.8 Western Blotting

Transfected cells were harvested 48 h after transfection as described above, washed once with PBS, and resuspended in 2x RotiLoad (Carl Roth) for lysis. The samples were sonified, and proteins were denatured at 95°C for 5 min. After separation on 12.5% SDS‐PAGE gels, proteins were transferred to PVDF membranes. Unspecific binding sites were blocked by incubation in 5% milk in TBS‐Tween for 30 min at room temperature, followed by incubation in primary antibody diluted in 2.5% milk in TBS‐Tween at 4°C overnight. Primary antibodies were ACSL4 (Santa Cruz, sc‐365230, 1:200), DMT1 (Abcam, ab55735, 1:4000), GPX4 (Abcam, ab125066, 1:1000), NCOA4 (Abcam, ab86707, 1:2000), and β‐Actin (Santa Cruz Biotechnology, sc‐47778, 1:400). Membranes were washed three times for 10 min before incubation in secondary antibody (dilution 1:7,500 in milk‐TBS‐Tween) for 1 h at room temperature. Western Lightning ECL reagent (Perkin Elmer) was used for chemiluminescence detection. Densiometric analysis of protein bands was performed with ImageJ software.

Validation statements, relevant citations, and antibody profiles are provided online by Santa Cruz Biotechnology and Abcam for each respective antibody used in this study.

### 4.9 Detection of Iron Levels via PhenGreen

Transfected cells were harvested 48 h after transfection as described above and seeded in 6‐well plates at a density of 500,000 cells per well. After 8 h incubation at 37°C, ferroptosis was induced by 1.5 µM IKE for 17–18.5 h. Cells were incubated with 5 µM PhenGreen SK Diacetate (Thermo Fisher Scientific) in PBS for 10 min at 37°C in the dark. For dead cell exclusion, 1 µg/mL propidium iodide (PI) was added to the cells 5 min before analysis on an Attune acoustic flow cytometer (Applied Biosystems). 15,000 PI‐negative events were recorded, and analysis was performed with FlowJo v10.8.1 Software (BD Life Sciences).

### 4.10 Detection of Iron Levels via ICP‐OES

Cells were seeded at a density of 125,000 cells per 10 cm dish and transfected as described above. After 24 h incubation at 37°C ferroptosis was induced by 2 µM IKE for 18 h. Cells were harvested and centrifuged at 300 x g for 5 min. The cell pellet was washed with 5 mL PBS twice. The cell count was counted via Vi‐CELL XR (Beckman Coulter) and adjusted to 1 Mio cells per sample in 1.5 mL ddH_2_O. Afterward, 1.5 mL of 65% nitric acid was added to the samples and incubated at room temperature overnight. Samples were stored at 4°C. Iron levels were measured via inductively coupled plasma optical emission spectrometry (ICP‐OES) using the Spectro Arcos 2 (Spectro Ametek).

### 4.11 Detection of Lipid Peroxidation

Cells were prepared as described for the detection of iron levels. However, for the analysis of lipid peroxidation 0.75 µM IKE was used with subsequent incubation at 37°C for 16.5‐19 h. Afterward BODIPY 581/591 C11 was added at a concentration of 2 µM followed by a further 30 min incubation at 37°C in the dark. Dead cell exclusion and analysis were performed as described above.

For 4‐HNE immunostaining, transfected cells were treated with 2 µM IKE 30 h after transfection and incubated at 37°C for 16.5‐19 h. Cells were harvested with 0.05% Trypsin‐EDTA, washed with PBS, and resuspended in 10% normal goat serum (Thermo Fisher Scientific) for 30 min on ice. Cells were incubated with anti‐4‐HNE antibody (Abcam, ab46545, 1:50) for 1 h on ice, washed three times with PBS, and incubated with anti‐rabbit Alexa 488 antibody (Thermo Fisher Scientific, A32731, 1:200) for 30 min on ice. After washing twice with PBS, cells were resuspended in PBS, and fluorescence was analyzed on an Attune acoustic flow cytometer (Applied Biosystems). Histograms and intensities were analyzed with FlowJo v10.8.1 Software (BD LifeSciences).

Validation statements, relevant citations, and antibody profiles are provided online by Thermo Fisher Scientific and Abcam for each respective antibody used in this study.

### 4.12 Detection of Glutathione Levels

HT‐1080 cells were seeded in 6‐well plates at a density of 150,000 cells per well and transfected as described above. After 48 h of incubation, 3 µM IKE was applied to the cells for 6 h before harvest. Equal numbers of cells per condition were lysed in PBS containing 0.5% Nonidet‐P40, and debris was removed by centrifugation (15 min, 20,000 x *g*, 4°C). The supernatant was transferred to a fresh tube, and proteins were removed using Deproteinizing Sample Preparation Kit—TCA (Abcam) according to the manufacturer's instructions. For the measurement of glutathione (GSH) levels, the fluorimetric GSH/GSSG Ratio Detection Assay Kit II (Abcam) was used with black µClear 96‐well plates with clear bottom. The preparation of standard curves and the assay procedure were performed according to the manufacturer's instructions. GSH levels were obtained by fluorescence detection with an EnVision 2104 Multilabel plate reader (PerkinElmer).

### 4.13 CRISPR Knockout Screen and Data Analysis

HT‐1080‐Cas9 cells were transduced with viruses containing the GeCKO v2 human CRISPR knockout pooled library [32] to generate the HT‐1080‐Cas9‐gRNA‐library cells. These cells were cultured in medium that was supplemented with 5 µg/mL of blasticidin and 0.7 µg/mL of puromycin. Next, the cells were expanded, and on day‐1, 18x T175 flasks were seeded at ∼20% confluency. By Day 3, when the cells reached ∼80% confluency, they were treated in duplicate as follows: DMSO (samples #1–2), IKE (0.8 µM, samples #3–4), and incubated for 20 h, then harvested on Day 4. The cells were washed once with PBS, detached using trypsin, and the sample duplicates were pooled. A 300 µL aliquot of each sample was diluted 1:1 with medium for viability assessment (ViCell). The remaining cells were washed twice with PBS (with centrifugation steps) and flash‐frozen on dry ice before storage at –80°C and submitted to the MSKCC GES Core.

Genomic DNA (gDNA) was isolated from cell pellets using standard extraction protocols, and the integrated guide RNA (gRNA) sequences were PCR‐amplified from the extracted gDNA using primers that flanked the gRNA cassette. The amplicons were then purified, quantified, and the resulting library was then subjected to next‐generation sequencing on an Illumina HiSeq platform to determine gRNA representation across the two different conditions (DMSO versus IKE). Normalized guide frequencies amplified from viable cells were scored against control cells to identify enriched or depleted guides using ENCoRE [43]. In some instances, a full complement of guides for each transcript was not present in amplified samples, and imputed values were utilized to calculate P‐values, False Discovery Rate (FDR), and fold changes. Results from the CRISPR KO screen are in Supplementary Table S3.

### 4.14 Analysis miRNA Integration

To identify target genes of the enriched miRNAs, we queried both the miRTarBase [44] and TargetScan [45] databases using default parameters. Consecutively, the resulting 95 putative validated target genes from miRTarBase were exploited as seed genes in a protein network analysis, performed using the Weighted Protein‐Protein Interaction (WPPI) Bioconductor package (Galhoz A, Turei D, P. Menden M (2022). wppi: Weighting protein‐protein interactions. R package version 1.6.0, https://github.com/aGalhoz/wppi) with default settings (i.e., without disease‐specific constraints besides the given seed genes). The top 10% highest scoring genes from WPPI were selected and integrated with the original miRNA target data from miRTarBase and TargetScan to expand the candidate gene list for downstream analyses.

### 4.15 RNA Sequencing Analysis

RNA was isolated from transfected cells, 48 h after transfection with InVitrap Spin Universal RNA Mini kit (Stratec Scientific) according to the manufacturer's instructions. 2.5 µg of each sample were shipped on dry ice to Genewiz (Azenta Life Sciences, Leipzig), where strand‐specific RNA sequencing (Illumina Nova‐Seq technology) with 20 million reads per sample and 2×150 bp read length was performed. Sample preparation included PolyA selection for rRNA removal. RNA sequencing data were subject to differential gene expression analysis leveraging the DESeq2 package and R software version 4.2.0. *P*‐values were estimated using Wald test and adjusted with Benjamini–Hochberg correction. Associations were considered significant at False Discovery Rate (FDR) of 1% and an absolute value of log2 fold‐change (log2FC) > 1. The raw counts are in Supplementary Table S4, and the differential expression is shown in Supplementary Table S5.

### 4.16 Proteomics Analysis

Transfected cells were harvested 48 h after transfection and resuspended in 300 µL RIPA buffer containing 1 mM PMSF. After incubation on ice for 15 min the samples were sonified at 50% amplitude three times for 2 pulses. After another 15 min on ice lysates were centrifuged (5 min at 14 000 x *g*) and the amount of protein in the supernatants was determined by Pierce BCA Protein Assay Kit (Thermo Fisher Scientific).

10 µg of whole cell extract were digested using a modified filter aided sample preparation (FASP) digest procedure [46]. After reduction and alkylation using DTT and IAA, the proteins were centrifuged on a 30 kDa cutoff filter device (Sartorius), washed thrice with UA buffer (8 M urea in 0.1 M Tris/HCl pH 8.5) and twice with 50 mM ammoniumbicarbonate. The proteins were digested for 2 h at room temperature using 0.5 µg Lys‐C (Wako Chemicals, Neuss, Germany) and for 16 h at 37°C using 1 µg trypsin (Promega). After centrifugation (10 min at 14 000 x *g*), the eluted peptides were acidified with 0.5% TFA and stored at ‐20°C.

LC‐MS/MS analysis was performed on a Q‐Exactive HF‐X mass spectrometer (Thermo Fisher Scientific) online coupled to an Ultimate 3000 nano‐RSLC (Thermo Fisher Scientific). Tryptic peptides were automatically loaded on a C18 trap column (300 µm inner diameter (ID) × 5 mm, Acclaim PepMap100 C18, 5 µm, 100 Å, LC Packings) at 30 µl/min flow rate prior to C18 reversed phase chromatography on the analytical column (nanoEase MZ HSS T3 Column, 100Å, 1.8 µm, 75 µm x 250 mm, Waters) at 250 nL/min flow rate in a 95 min non‐linear acetonitrile gradient from 3% to 40% in 0.1% formic acid. Profile precursor spectra from 300 to 1500 m/z were recorded at 60000 resolution with an automatic gain control (AGC) target of 3e6 and a maximum injection time of 30 ms TOP15 fragment spectra of charges 2 to 7 were recorded at 15000 resolution with an AGC target of 1e5, a maximum injection time of 50 ms, an isolation window of 1.6 m/z, a normalized collision energy of 28 and a dynamic exclusion of 30 s.

Generated raw files were analyzed using MaxQuant 2.0.3.1. (MPI Biochemistry, Martinsried) with default settings [47], additionally including LFQ quantification with LFQ min. ratio count of 1 [48], quantification using unique peptides, and application of the match‐between‐runs algorithm. Searches were performed within the MaxQuant software using the Andromeda search engine [49] against the Swissprot human protein database (20237 sequences, 11451954 residues) applying default search settings. Identifications were filtered for a peptide‐spectrum‐match and protein false discovery rate of each 1%. Missing values in the resulting list of identified protein groups were imputed from a normal distribution separately for each intensity column in Perseus (MPI Biochemistry, Martinsried) [50]. Resulting protein LFQ intensities were used for the calculation of fold‐changes and statistical values applying a Student's t‐test.

### 4.17 Lipidomics Analysis

Cells were seeded in 10‐cm culture dishes at a density of 2 Mio cells per dish. After 24 h, cells were transfected with hsa‐miR‐940 pre‐miR or pre‐miR negative control as described previously. Cells were treated with DMSO or 2 µM IKE for 18 h. Each condition was performed in five biological replicates. For lipidomics sample preparation, cells were harvested on dry ice to minimize metabolic activity. Culture dishes were washed twice with 11 mL ice‐cold PBS (dry ice), and all residual PBS was completely removed. Subsequently, 800 µL of ice‐cold 80% methanol (HPLC grade), prepared in glass flasks and stored at – 80°C overnight, was added directly onto the cells and rapidly distributed across the dish. Cells were scraped using a rubber‐tipped scraper, and the extract was transferred into collection tubes prepared on dry ice. Remaining cells were recovered with an additional 200 µL of 80% methanol and combined with the initial extract. Samples were stored at ‐80°C. After thawing the cell samples, about 80 mg of glass beads were added to each sample to break down the cells and release cellular contents during homogenization. Homogenization was conducted using Preccellys24 (PeqLab) for three cycles of 20 s at 5500 rpm, with a 30‐s break between cycles at 10°C. 200 µL of cell homogenate was then transferred into 1.5 mL glass vials for further extraction. Three replicates of 20 µL homogenates were pipetted in a 96‐well microtiter plate for Hoechst analysis [51].

Lipid extraction was performed by adding 60 µL water, 25 µL internal standard solution, and 575 µL methyl tert‐butyl ether (MTBE) to the sample homogenate in sequence, followed by incubation for 30 min on an orbital shaker DOS‐10L (Neolabline, Heidelberg, Germany) at 300 rpm. For phase separation, 200 µL of water was added to each vial. The mixtures were vortexed, and the vials were centrifuged at 5 000 x g for 10 min at RT with a Sigma 4–5C centrifuge (Qiagen, Hilden, Germany). The upper (organic) phase was transferred into two 1.5 mL glass vials, with 250 µL in each vial. The organic solvent was then evaporated under nitrogen gas using a TurboVap 96 dual evaporator (Biotage, Uppsala, Sweden). The aqueous phase was then reextracted by adding 100 µL methanol, 300 µL MTBE, and 100 µL water before the sample was incubated for 5 min at 300 rpm at room temperature. The sample was then centrifuged at 5 000 g for 10 min. The organic phase was transferred to the respective vials from the first extraction step (150 µL per vial) and evaporated to dryness under gaseous nitrogen. For quality control purposes, along with the biological samples, the pool of all homogenates, EDTA human reference plasma, and water were extracted using the same protocol as for the biological samples.

The dried extract samples were stored in ‐20°C until LC‐MS analysis. Prior to LC‐MS analysis, the dried extract was reconstituted in 40 µL of a mixture of 60% 2‐propanol/ 35% acetonitrile/ 5% water. Lipid analysis was performed using a Sciex ExionAD LC system coupled to a Sciex ZenoTOF 7600 (Sciex, Darmstadt, Germany). Lipids were separated as described by Witting et al. [52]. Briefly, a Waters Cortecs UPLC C18 column (150 mm x 2.1 mm ID, 1.6 µm particle size) was used with eluent A being 40% H2O/60% ACN + 10 mM ammonium formate / 0.1% form acid and eluent B being 10% ACN/90%iPrOH + 10 mM ammonium formate / 0.1% formic acid. Samples have been analyzed in positive and negative ionization modes using data‐dependent acquisition. Obtained raw data was processed using mzmine 4.8.30 [53], including conversion to. mzML format, peak picking, alignment, and annotation. Lipids were annotated using rule‐based lipid annotation in mzmine, and the results were exported as. csv files for further analysis in R and Excel. Data normalization based on Hoechst assay readouts and QC‐based intensity drift correction was performed in a custom R workflow using the notame package [54]. After normalization, only features present in all QC samples with an RSD < 30% were kept for further statistical analysis. For statistical analysis, Microsoft Excel (version 16.100) was used. The peak areas of the respective lipid features were log2 transformed and scaled relative to the mean values of all samples. A two‐tailed t‐test was performed to select significant (p ≤ 0.05) lipid changes and ferroptosis relevant lipids were chosen and presented in a heatmap created in the GraphPad Prism Software (version 10.6.1). Lipidomics data are in Supplementary Table S6.

### 4.18 Kaplan‐Meier Plotter

Ferroptosis‐sensitive cancer types, including sarcoma, liver hepatocellular carcinoma, and kidney renal papillary cell carcinoma, were analyzed for pan‐cancer survival analysis using the miRpower module of the Kaplan‐Meier Plotter (kmplot.com/mirpower) [37, 38, 39]. Patients were stratified based on hsa‐miR‐940 expression levels using a median split cut‐off. Kaplan‐Meier survival curves were generated to evaluate the association between miR‐940 expression and overall survival. Statistical significance was assessed using the log‐rank test, and hazard ratios (HR) with corresponding 95% confidence intervals were obtained to estimate the prognostic impact of miR‐940.

### 4.19 Statistical Analysis

Statistical analyses were performed using the GraphPad Prism Software (version 10.6.1). All relevant statistical information is provided in the respective figure legends.

## Author Contributions

K.H. and B.R.S. initiated the study; K.H. oversaw and supervised the whole study; A.K., J.T., S.A.I.W., D.K., K.K., L.R., S.S., and K.H. performed experiments and analyzed the data; M.F. and R.G. performed NGS of gDNA from the CRISPR KO screen; J.A.S. analyzed the CRISPR KO screen; A.G. and M.P.M. performed bioinformatics analyses and analyzed the transcriptomics data; A.A. and M.W. performed lipidomics, and M.W. and J.T. analyzed the data; J.M‐P. and S.M.H. performed proteomics and analyzed the data. M.W., H.Z., S.M.H., M.P.M., M.V., B.R.S., and K.H. supervised the work; S.A.I.W., A.K., and K.H. wrote the initial paper draft; All authors edited and finalized the paper.

## Conflicts of Interest

J.T. and K.H. are inventors on a patent involving ferroptosis. B.R.S. is an inventor on patents and patent applications involving ferroptosis; co‐founded and serves as a consultant to ProJenX, Inc., and serves as a consultant to Weatherwax Biotechnologies Corporation. The remaining authors declare no competing interests.

## Code Availability

Source code can be downloaded from https://github.com/MendenLab/miRNAInt_Ferroptosis.

## Responsible use of Artificial Intelligence

Artificial intelligence (AI) tools including ChatGPT, Gemini and DeepL were employed in this manuscript to refine grammar, spelling, and readability. These tools were used exclusively for linguistic enhancement, ensuring clarity and coherence without altering the scientific content, interpretation, or conclusions. All intellectual contributions, data analyses, and critical discussions remain the work of the authors. The final manuscript was thoroughly reviewed and approved by all co‐authors to ensure accuracy and integrity.

## Supporting information




**Supporting File 1**: advs75830‐sup‐0001‐SuppMat.pdf.

**Supporting File 2**: advs75830‐sup‐0002‐Suppl Table 1_Kolak_mir‐940 ferroptosis_sequence prediction.xlsx.

**Supporting File 3**: advs75830‐sup‐0003‐Suppl Table 2_Kolak_miR‐940 ferroptosis_qRT‐PCR primer.xlsx.

**Supporting File 4**: advs75830‐sup‐0004‐Suppl Table 3_Kolak_mir‐940 ferroptosis_CRISPR KO screen.xlsx.

**Supporting File 5**: advs75830‐sup‐0005‐Suppl Table 4_Kolak_mir‐940 ferroptosis_RNAseq_raw_counts.csv.

**Supporting File 6**: advs75830‐sup‐0006‐Suppl Table 5_Kolak_mir‐940 ferroptosis_DE RNA‐seq.xlsx.

**Supporting File 7**: advs75830‐sup‐0007‐Suppl Table 6_Kolak_mir‐940 ferroptosis_lipidomics full data.xlsx.

**Supporting File 8**: advs75830‐sup‐0008‐Legends for Supplementary Tables.docx.

## Data Availability

The raw data of this study are available from the corresponding author upon reasonable request. All results from CRISPR KO screen are in Supplementary Table S3. The RNAseq data (count data) generated in this study are provided in the Supplementary Table S4 and S5. Lipidomics data are in Supplementary Table S6. Proteomics data are deposited at PRIDE database (Project accession: PXD064621). Full western blots can be found in the supplementary (Supplementary Figure S6).

## References

[1] K. Hadian and B. R. Stockwell , “The Therapeutic Potential Of Targeting Regulated Non‐Apoptotic Cell Death,” Nature Reviews Drug Discovery 22, no. 9 (2023): 723–742, 10.1038/s41573-023-00749-8. 37550363

[2] S. J. Dixon , K. M. Lemberg , M. R. Lamprecht , et al., “Ferroptosis: An Iron‐Dependent Form of Nonapoptotic Cell Death,” Cell 149, no. 5 (2012): 1060–1072, 10.1016/j.cell.2012.03.042. 22632970 PMC3367386

[3] K. Hadian and B. R. Stockwell , “SnapShot: Ferroptosis,” Cell 181, no. 5 (2020): 1188–1188.e1, 10.1016/j.cell.2020.04.039. 32470402 PMC8157339

[4] B. R. Stockwell , “Ferroptosis Turns 10: Emerging Mechanisms, Physiological Functions, And Therapeutic Applications,” Cell 185, no. 14 (2022): 2401–2421, 10.1016/j.cell.2022.06.003. 35803244 PMC9273022

[5] J. Tschuck , V. Skafar , J. P. Friedmann Angeli , and K. Hadian , “The Metabolic Code Of Ferroptosis: Nutritional Regulators Of Cell Death,” Trends in Biochemical Sciences 50 (2025): p663–676. 10.1016/j.tibs.2025.04.007 40441931

[6] C. Berndt , H. Alborzinia , V. S. Amen , et al., “Ferroptosis in Health And Disease,” Redox Biology 75 (2024): 103211, 10.1016/j.redox.2024.103211. 38908072 PMC11253697

[7] H. Feng , K. Schorpp , J. Jin , et al., “Transferrin Receptor Is a Specific Ferroptosis Marker,” Cell Reports 30, no. 10 (2020): 3411–3423.e7, 10.1016/j.celrep.2020.02.049. 32160546 PMC7172030

[8] J. Jin , K. Schorpp , D. Samaga , K. Unger , K. Hadian , and B. R. Stockwell , “Machine Learning Classifies Ferroptosis and Apoptosis Cell Death Modalities With TfR1 Immunostaining,” ACS Chemical Biology 17, no. 3 (2022): 654–660, 10.1021/acschembio.1c00953. 35230809 PMC8938922

[9] K. Bersuker , J. M. Hendricks , Z. Li , et al., “The CoQ oxidoreductase FSP1 acts parallel to GPX4 to Inhibit Ferroptosis,” Nature 575, no. 7784 (2019): 688–692, 10.1038/s41586-019-1705-2. 31634900 PMC6883167

[10] S. Doll , F. P. Freitas , R. Shah , et al., “FSP1 is a Glutathione‐Independent Ferroptosis Suppressor,” Nature 575, no. 7784 (2019): 693–698, 10.1038/s41586-019-1707-0. 31634899

[11] E. Mishima , J. Ito , Z. Wu , et al., “A Non‐Canonical Vitamin K Cycle Is A Potent Ferroptosis Suppressor,” Nature 608, no. 7924 (2022): 778–783, 10.1038/s41586-022-05022-3. 35922516 PMC9402432

[12] V. A. N. Kraft , C. T. Bezjian , S. Pfeiffer , et al., “GTP Cyclohydrolase 1/Tetrahydrobiopterin Counteract Ferroptosis Through Lipid Remodeling,” ACS Central Science 6, no. 1 (2020): 41–53, 10.1021/acscentsci.9b01063. 31989025 PMC6978838

[13] M. Soula , R. A. Weber , O. Zilka , et al., “Metabolic Determinants Of Cancer Cell Sensitivity To Canonical Ferroptosis Inducers,” Nature Chemical Biology 16, no. 12 (2020): 1351–1360, 10.1038/s41589-020-0613-y. 32778843 PMC8299533

[14] J. Tschuck , W. Tonnus , S. Gavali , et al., “Seratrodast Inhibits Ferroptosis by Suppressing Lipid Peroxidation,” Cell Death & Disease 15, no. 11 (2024): 853, 10.1038/s41419-024-07251-y. 39578434 PMC11584883

[15] J. Tschuck , L. Theilacker , I. Rothenaigner , et al., “Farnesoid X Receptor Activation By Bile Acids Suppresses Lipid Peroxidation And Ferroptosis,” Nature Communications 14, no. 1 (2023): 6908, 10.1038/s41467-023-42702-8. PMC10616197 37903763

[16] J. Tschuck , V. P. Nair , A. Galhoz , et al., “Suppression of Ferroptosis By Vitamin A Or Radical‐Trapping Antioxidants Is Essential For Neuronal Development,” Nature Communications 15, no. 1 (2024): 7611, 10.1038/s41467-024-51996-1. PMC11366759 39218970

[17] D. Liang , Y. Feng , F. Zandkarimi , et al., “Ferroptosis Surveillance Independent of GPX4 and Differentially Regulated By Sex Hormones,” Cell 186, no. 13 (2023): 2748–2764.e22, 10.1016/j.cell.2023.05.003. 37267948 PMC10330611

[18] W. Tonnus , F. Maremonti , S. Gavali , et al., “Multiple Oestradiol Functions Inhibit Ferroptosis And Acute Kidney Injury,” Nature 645, no. 8082 (2025): 1011–1019, 10.1038/s41586-025-09389-x. 40804518 PMC12460175

[19] A. M. Mohr and J. L. Mott , “Overview of MicroRNA Biology,” Seminars in Liver Disease 35, no. 1 (2015): 003–011, 10.1055/s-0034-1397344. PMC4797991 25632930

[20] A. Balihodzic , F. Prinz , M. A. Dengler , G. A. Calin , P. J. Jost , and M. Pichler , “Non‐coding RNAs and Ferroptosis: Potential Implications For Cancer Therapy,” Cell Death & Differentiation 29, no. 6 (2022): 1094–1106, 10.1038/s41418-022-00998-x. 35422492 PMC9177660

[21] H. Li , Y. Li , D. Tian , J. Zhang , and S. Duan , “miR‐940 is a New Biomarker With Tumor Diagnostic And Prognostic Value,” Molecular Therapy—Nucleic Acids 25 (2021): 53–66, 10.1016/j.omtn.2021.05.003. 34168918 PMC8192490

[22] P. Li , Z. Xiao , J. Luo , Y. Zhang , and L. Lin , “MiR‐139‐5p, miR‐940 and miR‐193a‐5p Inhibit The Growth Of Hepatocellular Carcinoma By Targeting SPOCK1,” Journal of Cellular and Molecular Medicine 23, no. 4 (2019): 2475–2488, 10.1111/jcmm.14121. 30710422 PMC6433657

[23] K. Jiang , T. Zhao , M. Shen , et al., “MiR‐940 inhibits TGF‐β‐Induced Epithelial‐Mesenchymal Transition And Cell Invasion By Targeting Snail In Non‐Small Cell Lung Cancer,” Journal of Cancer 10, no. 12 (2019): 2735–2744, 10.7150/jca.31800. 31258781 PMC6584929

[24] X. Liu , X. Ge , Z. Zhang , et al., “MicroRNA‐940 Promotes Tumor Cell Invasion And Metastasis By Downregulating ZNF24 in Gastric Cancer,” Oncotarget 6, no. 28 (2015): 25418–25428, 10.18632/oncotarget.4456. 26317898 PMC4694841

[25] K. Su , C. F. Wang , Y. Zhang , Y. J. Cai , Y. Y. Zhang , and Q. Zhao , “miR‐940 Upregulation Contributes To Human Cervical Cancer Progression Through p27 and PTEN Inhibition,” International Journal of Oncology 50, no. 4 (2017): 1211–1220, 10.3892/ijo.2017.3897. 28350106

[26] J. Zhao , Y. Hou , C. Yin , et al., “Upregulation of Histamine Receptor H1 Promotes Tumor Progression And Contributes To Poor Prognosis In Hepatocellular Carcinoma,” Oncogene 39, no. 8 (2020): 1724–1738, 10.1038/s41388-019-1093-y. 31740780 PMC7033043

[27] R. Xu , F. Zhou , T. Yu , et al., “MicroRNA‐940 Inhibits Epithelial‐Mesenchymal Transition Of Glioma Cells Via Targeting ZEB2,” American journal of translational research 11, no. 12 (2019): 7351. 31934283 PMC6943459

[28] D. Sun , L. Chen , H. Lv , Y. Gao , X. Liu , and X. Zhang , “Circ_0058124 Upregulates MAPK1 Expression to Promote Proliferation, Metastasis and Metabolic Abilities in Thyroid Cancer Through Sponging miR‐940,” OncoTargets and Therapy 13 (2020): 1569–1581, 10.2147/OTT.S237307. 32110054 PMC7037104

[29] S. Wang , L. Xu , X. Che , et al., “E3 Ubiquitin Ligases Cbl‐b and c‐Cbl Downregulate PD ‐L1 in EGFR Wild‐Type Non‐Small Cell Lung Cancer,” FEBS Letters 592, no. 4 (2018): 621–630, 10.1002/1873-3468.12985. 29364514

[30] J. Ma , F. Sun , C. Li , et al., “Depletion of Intermediate Filament Protein Nestin, a target Of MicroRNA‐940, Suppresses Tumorigenesis By Inducing Spontaneous DNA Damage Accumulation In Human Nasopharyngeal Carcinoma,” Cell Death & Disease 5, no. 8 (2014): 1377, 10.1038/cddis.2014.293. PMC4454294 25118937

[31] T. Xu , K. Zhang , J. Shi , et al., “MicroRNA‐940 Inhibits Glioma Progression By Blocking Mitochondrial Folate Metabolism Through Targeting of MTHFD2,” American Journal of Cancer Research 9, no. 2 (2019): 250. 30906627 PMC6405966

[32] O. Shalem , N. E. Sanjana , E. Hartenian , et al., “Genome‐Scale CRISPR‐Cas9 Knockout Screening in Human Cells,” Science 343, no. 6166 (2014): 84–87, 10.1126/science.1247005. 24336571 PMC4089965

[33] Y. Zhang , H. Tan , J. D. Daniels , et al., “Imidazole Ketone Erastin Induces Ferroptosis and Slows Tumor Growth in a Mouse Lymphoma Model,” Cell Chemical Biology 26, no. 5 (2019): 623–633.e9, 10.1016/j.chembiol.2019.01.008. 30799221 PMC6525071

[34] H. Wang , T. Song , Y. Qiao , and J. Sun , “miR940 Inhibits Cell Proliferation And Promotes Apoptosis In Esophageal Squamous Cell Carcinoma Cells And Is Associated With PostOperative Prognosis,” Experimental and therapeutic medicine 19, no. 2 (2020): 833. 32010243 10.3892/etm.2019.8279 PMC6966135

[35] C. Yu , C. Zhang , L. Zhang , J. Gu , and L. Qin , “MicroRNA‐940 induces Apoptosisof HTR8‐S/Vneo Cells in Intrahepatic Cholestasis of Pregnancy by Regulating RhoA,”The Tohoku Journal of Experimental Medicine 268 (2025): 419–426. 40967808 10.1620/tjem.2025.J104

[36] Y. Fan , X. Che , K. Hou , et al., “MiR‐940 Promotes The Proliferation And Migration Of Gastric Cancer Cells Through Up‐Regulation Of Programmed Death Ligand‐1 Expression,” Experimental Cell Research 373, no. 1‐2 (2018): 180–187, 10.1016/j.yexcr.2018.10.011. 30367831

[37] B. Győrffy , “Transcriptome‐Level Discovery Of Survival‐Associated Biomarkers And Therapy Targets In Non‐Small‐Cell Lung Cancer,” British Journal of Pharmacology 181, no. 3 (2024): 362–374, 10.1111/bph.16257. 37783508

[38] B. Győrffy , “Integrated Analysis Of Public Datasets For The Discovery And Validation Of Survival‐Associated Genes In Solid Tumor,” Innovation (Camb) 5, no. 3 (2024): 100625. 38706955 10.1016/j.xinn.2024.100625 PMC11066458

[39] A. Lánczky and B. Győrffy , “Web‐Based Survival Analysis Tool Tailored for Medical Research (KMplot): Development and Implementation,” Journal of Medical Internet Research 23, no. 7 (2021): 27633, 10.2196/27633. PMC8367126 34309564

[40] S. Kishore , A. R. Gruber , D. J. Jedlinski , A. P. Syed , H. Jorjani , and M. Zavolan , “Insights Into snoRNA Biogenesis and Processing From PAR‐CLIP of snoRNA Core Proteins and Small RNA Sequencing,” Genome Biology 14, no. 5 (2013): R45, 10.1186/gb-2013-14-5-r45. 23706177 PMC4053766

[41] S. Doll , B. Proneth , Y. Y. Tyurina , et al., “ACSL4 Dictates Ferroptosis Sensitivity By Shaping Cellular Lipid Composition,” Nature Chemical Biology 13, no. 1 (2017): 91–98, 10.1038/nchembio.2239. 27842070 PMC5610546

[42] K. Hadian and B. R. Stockwell , “A Roadmap To Creating Ferroptosis‐Based Medicines,” Nature Chemical Biology 17, no. 11 (2021): 1113–1116, 10.1038/s41589-021-00853-z. 34675413 PMC8990224

[43] D. Trümbach , S. Pfeiffer , M. Poppe , et al., “ENCoRE: an Efficient Software for CRISPR Screens Identifies New Players In Extrinsic Apoptosis,” BMC Genomics 18, no. 1 (2017): 905, 10.1186/s12864-017-4285-2. 29178829 PMC5702081

[44] H. Y. Huang , Y. C. Lin , S. Cui , et al., “miRTarBase update 2022: an Informative Resource For Experimentally Validated miRNA–Target Interactions,” Nucleic Acids Research 50, no. D1 (2022): D222–D230, 10.1093/nar/gkab1079. 34850920 PMC8728135

[45] S. E. McGeary , K. S. Lin , C. Y. Shi , et al., “The Biochemical Basis of microRNA Targeting Efficacy,” Science 366, no. 6472 (2019): aav1741. 10.1126/science.aav1741 PMC7051167 31806698

[46] J. R. Wiśniewski , A. Zougman , N. Nagaraj , and M. Mann , “Universal Sample Preparation Method For Proteome Analysis,” Nature Methods 6, no. 5 (2009): 359–362, 10.1038/nmeth.1322. 19377485

[47] S. Tyanova , T. Temu , and J. Cox , “The MaxQuant Computational Platform For Mass Spectrometry‐Based Shotgun Proteomics,” Nature Protocols 11, no. 12 (2016): 2301–2319, 10.1038/nprot.2016.136. 27809316

[48] J. Cox , M. Y. Hein , C. A. Luber , I. Paron , N. Nagaraj , and M. Mann , “Accurate Proteome‐wide Label‐free Quantification by Delayed Normalization and Maximal Peptide Ratio Extraction, Termed MaxLFQ,” Molecular & Cellular Proteomics 13, no. 9 (2014): 2513–2526, 10.1074/mcp.M113.031591. 24942700 PMC4159666

[49] J. Cox , N. Neuhauser , A. Michalski , R. A. Scheltema , J. V. Olsen , and M. Mann , “Andromeda: A Peptide Search Engine Integrated Into the MaxQuant Environment,” Journal of Proteome Research 10, no. 4 (2011): 1794–1805, 10.1021/pr101065j. 21254760

[50] S. Tyanova , T. Temu , P. Sinitcyn , et al., “The Perseus Computational Platform For Comprehensive Analysis Of (Prote)Omics Data,” Nature Methods 13, no. 9 (2016): 731–740, 10.1038/nmeth.3901. 27348712

[51] C. Muschet , G. Möller , C. Prehn , M. H. de Angelis , J. Adamski , and J. Tokarz , “Removing the Bottlenecks Of Cell Culture Metabolomics: Fast Normalization Procedure, Correlation Of Metabolites To Cell Number, And Impact Of The Cell Harvesting Method,” Metabolomics 12, no. 10 (2016): 151, 10.1007/s11306-016-1104-8. 27729828 PMC5025493

[52] M. Witting , T. V. Maier , S. Garvis , and P. Schmitt‐Kopplin , “Optimizing a ultrahigh Pressure Liquid Chromatography‐Time Of Flight‐Mass Spectrometry Approach Using A Novel Sub‐2 µm Core–Shell Particle For In Depth Lipidomic Profiling Of Caenorhabditis Elegans,” Journal of Chromatography A 1359 (2014): 91–99, 10.1016/j.chroma.2014.07.021. 25074420

[53] R. Schmid , S. Heuckeroth , A. Korf , et al., “Integrative Analysis Of Multimodal Mass Spectrometry Data in MZmine 3,” Nature Biotechnology 41, no. 4 (2023): 447–449, 10.1038/s41587-023-01690-2. PMC10496610 36859716

[54] A. Klåvus , M. Kokla , S. Noerman , et al., “Notame′: Workflow for Non‐Targeted LC–MS Metabolic Profiling,” Metabolites 10, no. 4 (2020): 135. 32244411 10.3390/metabo10040135 PMC7240970

