# Supplementary material for: MiR‐940 Suppresses Ferroptosis by Controlling Expression of Key Regulatory Genes
Source: Adv Sci (Weinh). 2026 May 29;13(46):e75830. doi: 10.1002/advs.75830 (PMC13336029; doi:10.1002/advs.75830)
Supplement: Supplementary file 1 [file ADVS-13-e75830-s002.pdf]

Supplementary Figures

for

miR-940 suppresses ferroptosis by controlling expression of key regulatory genes

Andrea Kolak[1,§], Juliane Tschuck[1,§], Stefanie A. I. Weiß[1,§], Daniel Kaemena[2,§], Karolin Klimm[1], Ana Galhoz[3], Larissa Ringelstetter[1,4], Myles Fennell[5], Juliane Merl-Pham[6], Anna Artati[6], Stefanie Strasser[1], Ralph Garippa[5], Michael Witting[6,7], Hans Zischka[8,9], Joel A. Schick[10], Stefanie M. Hauck[6], Michael P. Menden[3,11], Michelle Vincendeau[2,12], Brent R. Stockwell[4,13], and Kamyar Hadian[1,4,#]

[1] Cell Signaling and Chemical Biology, Research Unit Signaling and Translation, Helmholtz Zentrum München, Neuherberg, Germany

[2] Institute of Virology, Helmholtz Zentrum München, Neuherberg, Germany.

[3] Computational Health Center, Helmholtz Zentrum München, Neuherberg, Germany.

[4] Department of Biological Sciences, Columbia University, New York, NY, USA

[5] Gene Editing & Screening Core Facility, Memorial Sloan Kettering Cancer Center, New York, NY, USA

[6] Metabolomics and Proteomics Core, Helmholtz Zentrum München, Neuherberg, Germany

[7] Chair of Analytical Food Chemistry, TUM School of Life Sciences, Technical University of Munich, Freising-Weihenstephan, Germany

[8] Institute of Molecular Toxicology and Pharmacology, Helmholtz Zentrum München, Neuherberg, Germany

[9] Institute of Toxicology and Environmental Hygiene, Technical University Munich, School of Medicine and Health, Munich, Germany

[10] Genetics and Cellular Engineering Group, Research Unit Signaling and Translation, Helmholtz Zentrum Munich, Neuherberg, Germany

[11] Department of Biochemistry and Pharmacology, Bio21 Molecular Science and Biotechnology Institute, The University of Melbourne, Parkville, Victoria, Australia

[12] Institute of Virology, School of Medicine and Health, Technical University Munich, Germany

[13] Department of Chemistry, Department of Pathology and Cell Biology, Herbert Irving Comprehensive Cancer Center, Irving Institute for Cancer Dynamics, Data Science Institute, Digestive and Liver Disease Research Center, Columbia University, New York, NY, USA

[§] These authors contributed equally to this work

[#] Correspondence: Kamyar Hadian, kamyar.hadian@helmholtz-munich.de

Supplementary Figure 1

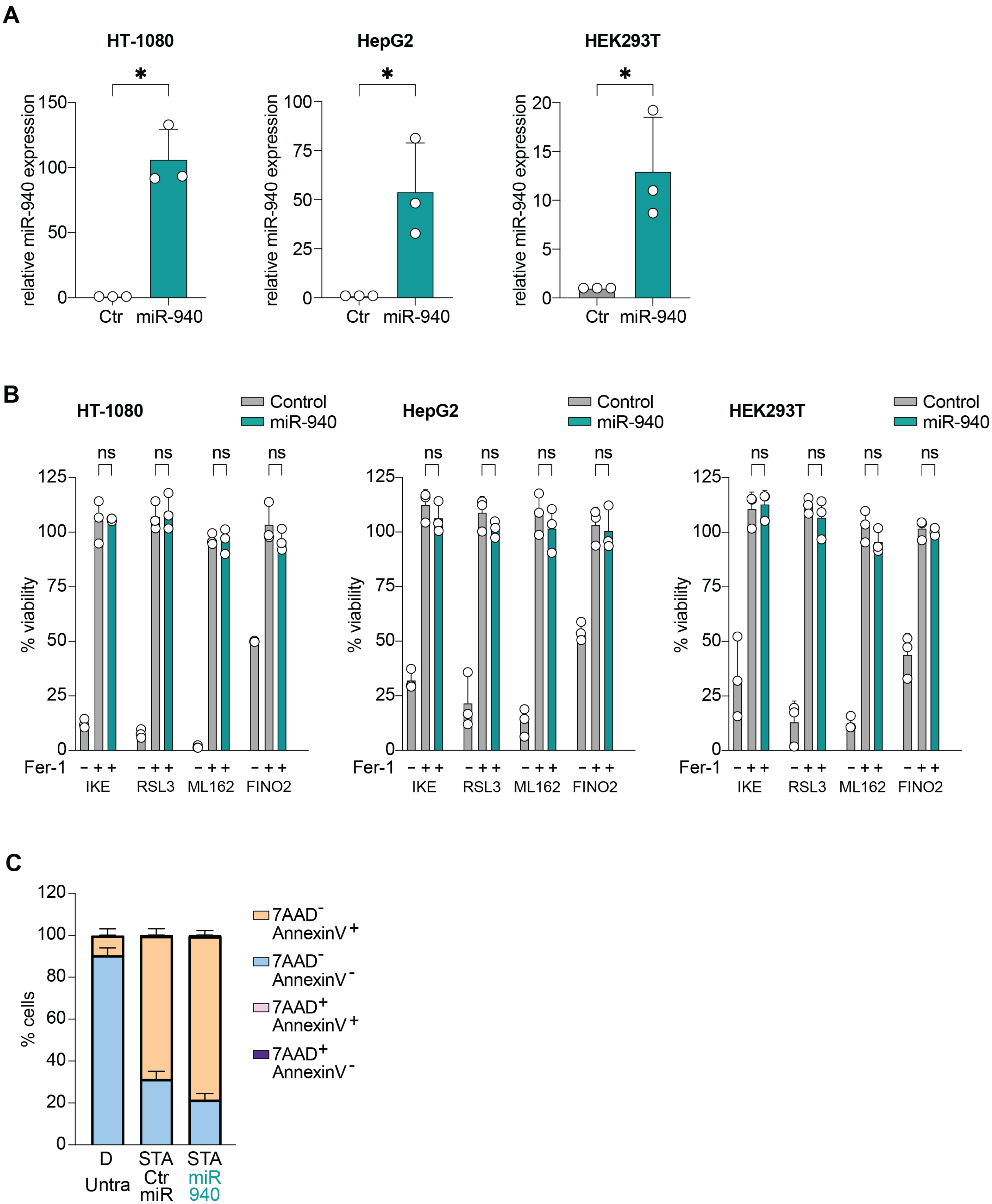

Supplementary Figure 1: miR-940 inhibits IKE-induced ferroptosis

A) Relative miR-940-expression in pre-miR-940- and control-transfected HT-1080, HepG2 and HEK293T cells, measured with qRT-PCR, * = p ≤ 0.05, unpaired t-test with Welch's correction, data are mean ± SD of n = 3 biological replicates. **B)** Inhibition of ferroptosis in pre-miR-940 and control transfected HT-1080, HepG2, HEK293T treated with 0.3 µM IKE, 0.25 µM RSL3, 0.25 µM ML162 and 0.6 µM FINO2 (HT-1080), 2.5 µM IKE, 0.5 µM RSL3, 0.25 µM ML162 and 2,5 µM FINO2 (HepG2), 0.6 µM IKE, 0.25 µM RSL3, 0.25 µM ML162 and 1.25 µM FINO2 (HEK293T) with 2 µM Fer-1, ns = p > 0.05, 2way ANOVA, data are mean ± SD of n = 3 biological replicates. **C)** 7-AAD/AnnexinV-Staining in pre-miR-940- , control-transfected and untransfected HT-1080 cells 24h post transfection treated with Staurosporine (STA, 1 µM), data are mean ± SD of n = 3 biological replicates.

Supplementary Figure 2

- **TargetScan**:
  - <u>*NCOA4*:</u> predicted pairing at position 117-124 of the 3'UTR (RNA sequence **CCUGCCUA**, hsa-miR-940 sequence **GGACGGAA**).

<table>
<tr><th></th><th>Predicted consequential pairing of target region (top) and miRNA (bottom)</th><th>Site type</th><th>Context++ score</th><th>Context++ score percentile</th><th>Weighted context++ score</th><th>Conserved branch length</th><th>P_{CT}</th><th>Predicted relative K_D</th></tr>
<tr><td>Position 117-124 of NCOA4 3' UTR
hsa-miR-6893-5p</td><td>5' ...UAGCUUAGUUCUUCUCCUGCCUA...
|||||||
3' CGAGGUGGGAUGUGGACGGAC</td><td>8mer</td><td>-0.37</td><td>99</td><td>-0.37</td><td>0.075</td><td>N/A</td><td>N/A</td></tr>
<tr><td>Position 117-124 of NCOA4 3' UTR
hsa-miR-6808-5p</td><td>5' ...UAGCUUAGUUCUUCUCCUGCCUA...
|||||||
3' GUACCAGGGUGGAGGGACGGAC</td><td>8mer</td><td>-0.37</td><td>99</td><td>-0.37</td><td>0.075</td><td>N/A</td><td>N/A</td></tr>
<tr><td>Position 117-124 of NCOA4 3' UTR
hsa-miR-940</td><td>5' ...UAGCUUAGUUCUUCUCCUGCCUA...
|||||||
3' CCCCUCGCCCCCGGGACGGAA</td><td>8mer</td><td>-0.37</td><td>99</td><td>-0.37</td><td>0.075</td><td>N/A</td><td>N/A</td></tr>
</table>

  - <u>*ACSL4*:</u> predicted pairing at position 2272-2278 of the 3'UTR (RNA sequence **CCUGCCU**, hsa-miR-940 sequence **GGACGGA**).

<table>
<tr><th></th><th>Predicted consequential pairing of target region (top) and miRNA (bottom)</th><th>Site type</th><th>Context++ score</th><th>Context++ score percentile</th><th>Weighted context++ score</th><th>Conserved branch length</th><th>P_{CT}</th><th>Predicted relative K_D</th></tr>
<tr><td>Position 2272-2278 of ACSL4 3' UTR
hsa-miR-6893-5p</td><td>5' ...AAUUCCAUGAAUGUUCCUGCCUC...
|||||||
3' CGAGGUGGGAUGUGGACGGAC</td><td>7mer-m8</td><td>-0.18</td><td>91</td><td>-0.10</td><td>0.043</td><td>N/A</td><td>N/A</td></tr>
<tr><td>Position 2272-2278 of ACSL4 3' UTR
hsa-miR-6808-5p</td><td>5' ...AAUUCCAUGAAUGUUCCUGCCUC...
|||||||
3' GUACCAGGGUGGAGGGACGGAC</td><td>7mer-m8</td><td>-0.17</td><td>89</td><td>-0.09</td><td>0.043</td><td>N/A</td><td>N/A</td></tr>
<tr><td>Position 2272-2278 of ACSL4 3' UTR
hsa-miR-940</td><td>5' ...AAUUCCAUGAAUGUUCCUGCCUC...
|||||||
3' CCCCUCGCCCCCGGGACGGAA</td><td>7mer-m8</td><td>-0.14</td><td>86</td><td>-0.08</td><td>0.043</td><td>N/A</td><td>N/A</td></tr>
</table>

  - <u>*LPCAT3*:</u> two predicted pairings at positions 273-279 and 650-656 of the 3'UTR (RNA sequence **CCUGCCU**, hsa-miR-940 sequence **GGACGGA**).

<table>
<tr><th></th><th>Predicted consequential pairing of target region (top) and miRNA (bottom)</th><th>Site type</th><th>Context++ score</th><th>Context++ score percentile</th><th>Weighted context++ score</th><th>Conserved branch length</th><th>P_{CT}</th><th>Predicted relative K_D</th></tr>
<tr><td>Position 273-279 of LPCAT3 3' UTR
hsa-miR-940</td><td>5' ...GGUUCAAGUGAUUUUCCUGCCUC...
|||||||
3' CCCCUCGCCCCCGGGACGGAA</td><td>7mer-m8</td><td>-0.20</td><td>94</td><td>-0.20</td><td>0.073</td><td>N/A</td><td>N/A</td></tr>
<tr><td>Position 273-279 of LPCAT3 3' UTR
hsa-miR-6893-5p</td><td>5' ...GGUUCAAGUGAUUUUCCUGCCUC...
|||||||
3' CGAGGUGGGAUGUGGACGGAC</td><td>7mer-m8</td><td>-0.20</td><td>93</td><td>-0.20</td><td>0.073</td><td>N/A</td><td>N/A</td></tr>
<tr><td>Position 273-279 of LPCAT3 3' UTR
hsa-miR-6808-5p</td><td>5' ...GGUUCAAGUGAUUUUCCUGCCUC...
|||||||
3' GUACCAGGGUGGAGGGACGGAC</td><td>7mer-m8</td><td>-0.19</td><td>92</td><td>-0.19</td><td>0.073</td><td>N/A</td><td>N/A</td></tr>
<tr><td>Position 650-656 of LPCAT3 3' UTR
hsa-miR-940</td><td>5' ...AGAGGGGUGCAAGGCCCUGCCUG...
|||||||
3' CCCCUCGCCCCCGGGACGGAA</td><td>7mer-m8</td><td>-0.05</td><td>66</td><td>-0.05</td><td>0.073</td><td>N/A</td><td>N/A</td></tr>
</table>

  - <u>*DMT1*</u>: predicted pairing at position 1433-1439 of the 3'UTR (RNA sequence **CCUGCCU**, hsa-miR-940 sequence **GGACGGA**).

<table>
<tr><th></th><th>Predicted consequential pairing of target region (top) and miRNA (bottom)</th><th>Site type</th><th>Context++ score</th><th>Context++ score percentile</th><th>Weighted context++ score</th><th>Conserved branch length</th><th>P_{CT}</th><th>Predicted relative K_D</th></tr>
<tr><td>Position 1433-1439 of SLC11A2 3' UTR
hsa-miR-940</td><td>5' ...ACCUCAAGUGAUCUACCUGCCUC...
|||||||
3' CCCCUCGCCCCCGGGACGGAA</td><td>7mer-m8</td><td>-0.12</td><td>83</td><td>0.00</td><td>0.099</td><td>N/A</td><td>N/A</td></tr>
</table>

  - <u>*GPX4*: </u>predicted pairing at position 98-104 of the 3'UTR (RNA sequence **CCUGCCU**, hsa-miR-940 sequence **GGACGGA**).

<table>
<tr><th></th><th>Predicted consequential pairing of target region (top) and miRNA (bottom)</th><th>Site type</th><th>Context++ score</th><th>Context++ score percentile</th><th>Weighted context++ score</th><th>Conserved branch length</th><th>P_{CT}</th><th>Predicted relative K_D</th></tr>
<tr><td>Position 98-104 of GPX4 3' UTR
hsa-miR-940</td><td>5' ...CGGCACUCAUGACGGCCUGCCUG...
|||||||
3' CCCCUCGCCCCCGGGACGGAA</td><td>7mer-m8</td><td>-0.25</td><td>97</td><td>-0.25</td><td>0.016</td><td>N/A</td><td>N/A</td></tr>
</table>

- **miRDB**: We identified one high-confidence site in *NCOA4* (8-mer, score = 84 out of 100), convergent with the TargetScan prediction at the same 3'UTR position (117-124).

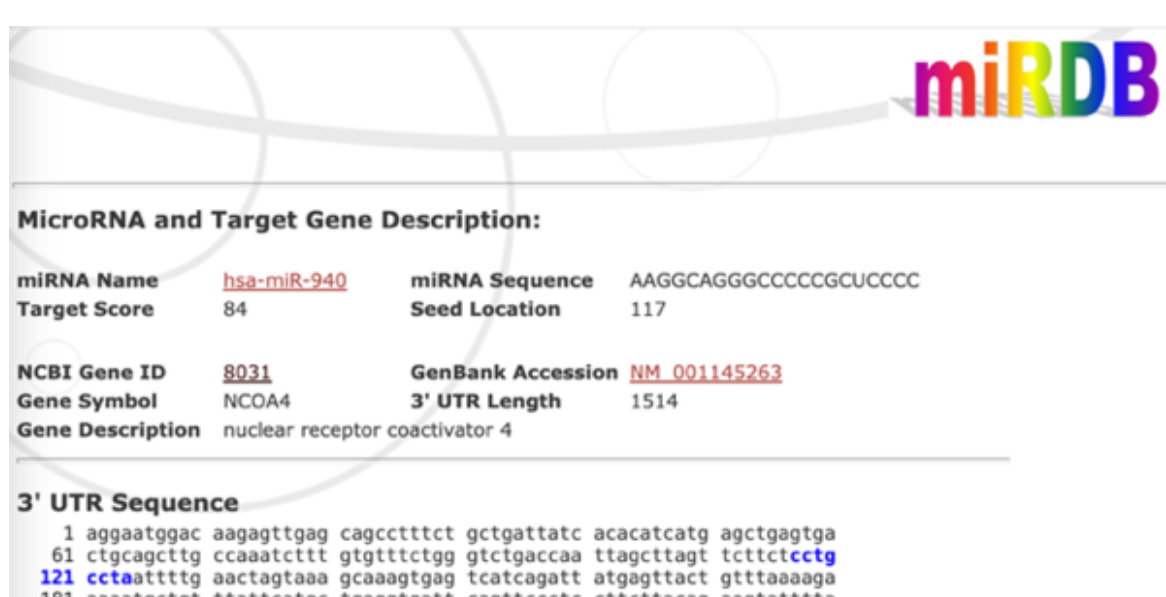

- **miRTarBase**: We identified a validated functional interaction between *DMT1* and miR-940 in the 3'UTR.

Supplementary Figure 2: Prediction of direct binding sites for mature hsa-miR-940 sequence
Prediction of direct binding sites for mature hsa-miR-940 sequence from miRbase as well as evaluated predicted binding sites using TargetScan (predicted targets), miRDB (predicted targets), and miRTarBase (validated targets). TargetScan predicted pairing of targets NCOA4, ACSL4, LPCAT3, DMT1 and GPX4. In addition, the miRDB identified the target NCOA4 and the miRTarBase the target DMT1.

Supplementary Figure 3

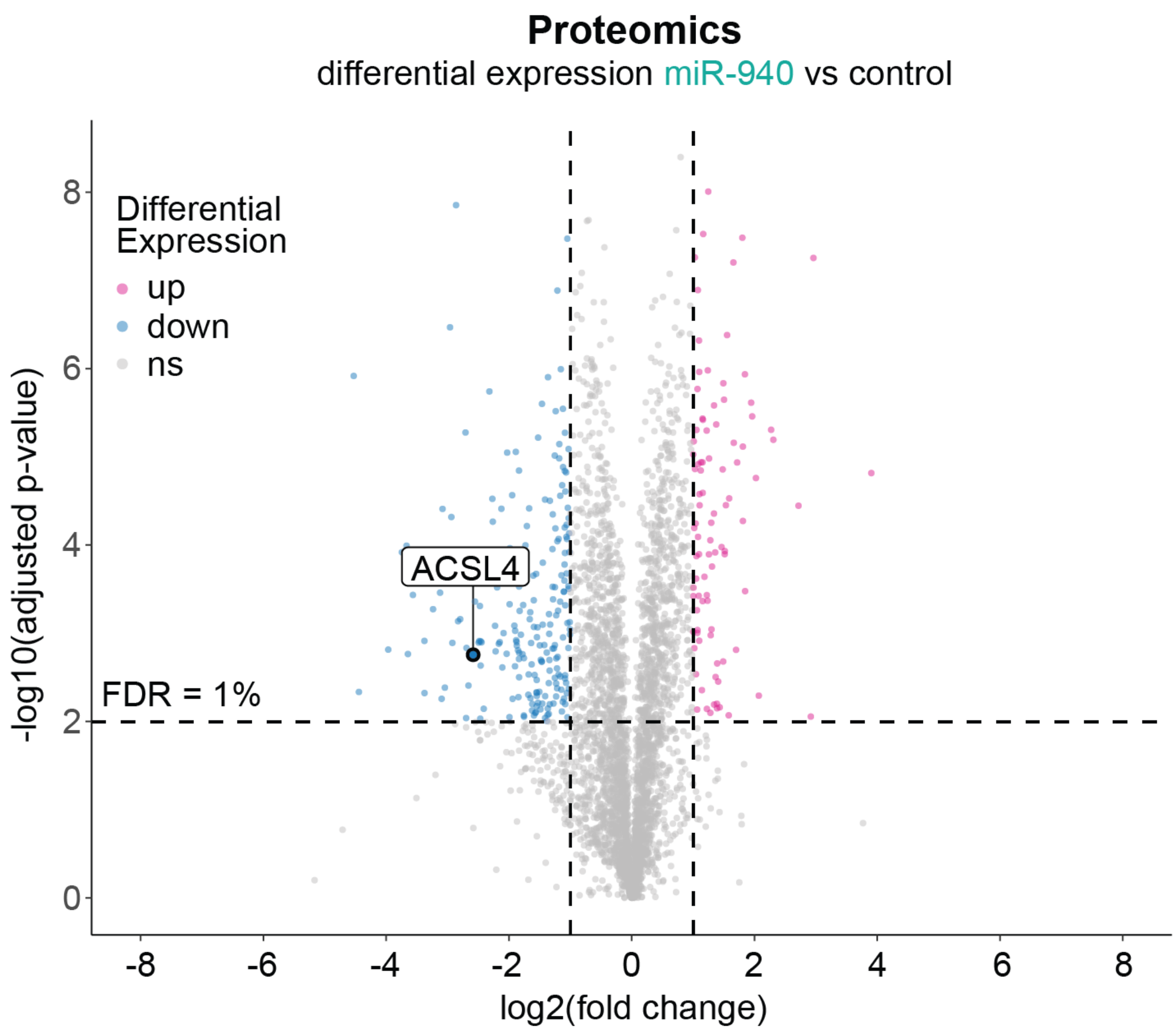

Supplementary Figure 3: Proteomics analysis of miR-940 transfected cells
Proteomics measurements of HT-1080 cells transfected with pre-miR-940 show downregulation of ACSL4 compared to control cells, FDR of 1%, Student's t-test.

Supplementary Figure 4

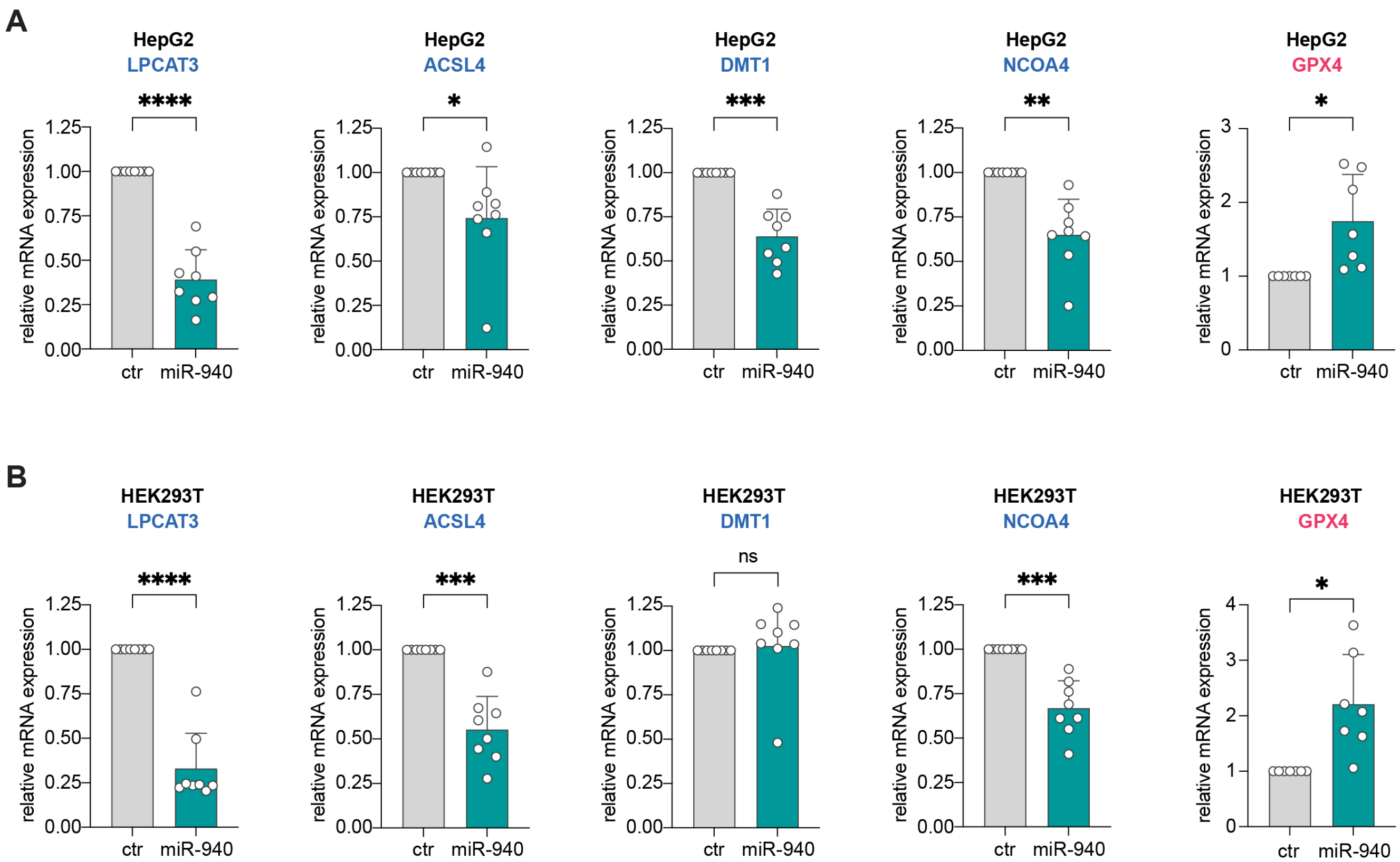

Supplementary Figure 4: miR-940 modulates key regulators of ferroptotic cell death in HepG2 and HEK293T cells
A,B) qRT-PCR of LPCAT3, ACSL4, DMT1, NCOA4, and GPX4 mRNA levels after pre-miR-940 transfection in HepG2 (A) and HEK293T (B) cells; ns = $p > 0.05$, * = $p \leq 0.05$, ** = $p \leq 0.01$ 2way ANOVA, data are mean ± SD of (A) n = 3 biological replicates and (B) 5 biological replicates.

Supplementary Figure 5

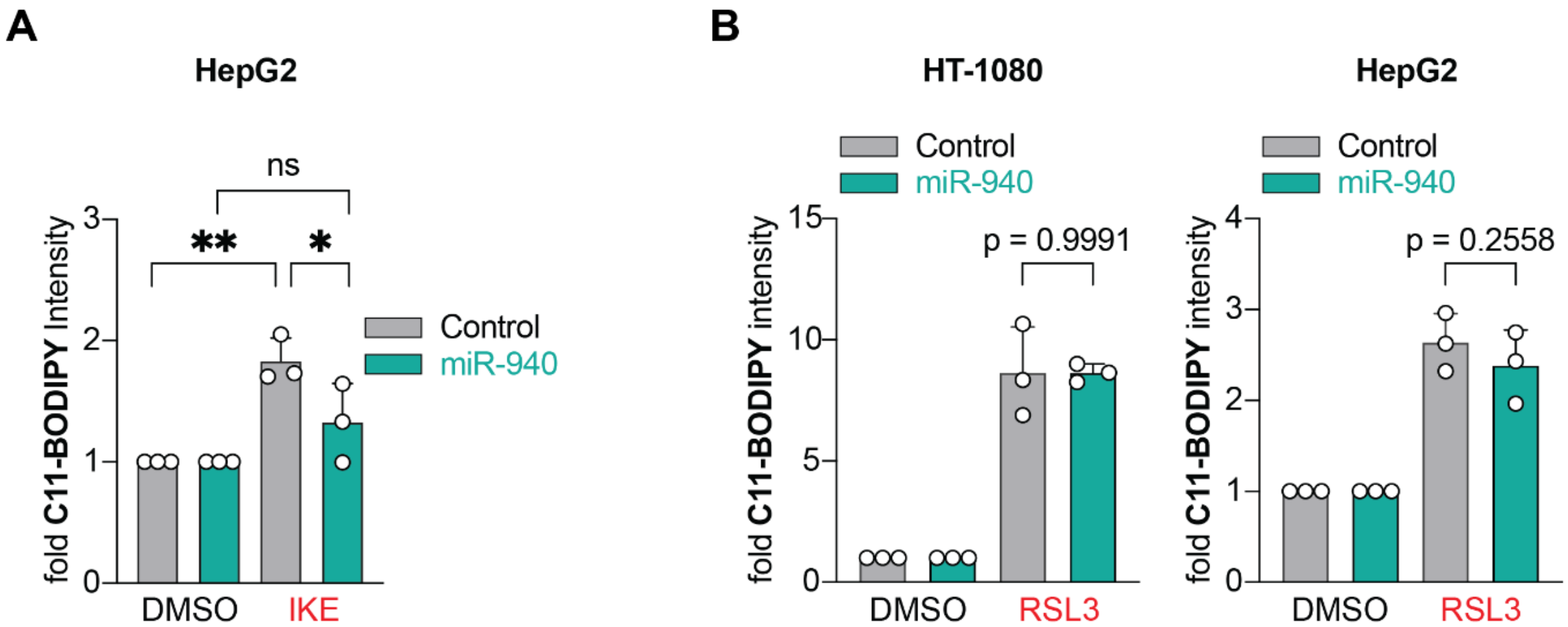

Supplementary Figure 5: miR-940 suppresses lipid peroxidation
A) C11-BODIPY sensor of lipid peroxidation upon treatment with 2 µM IKE in miR-940 overexpressing HepG2 cells, ns = p > 0.05, * = p ≤ 0.05, ** = p ≤ 0.01 2way ANOVA, data are mean ± SD of n = 3 biological replicates. **B)** C11-BODIPY sensor of lipid peroxidation upon treatment with 150 nM RSL3 in miR-940 overexpressing HT-1080 cells as well as 1 µM RSL3 in miR-940 overexpressing HepG2 cells; 2way ANOVA, data are mean ± SD of n = 3 biological replicates.

Supplementary Figure 6

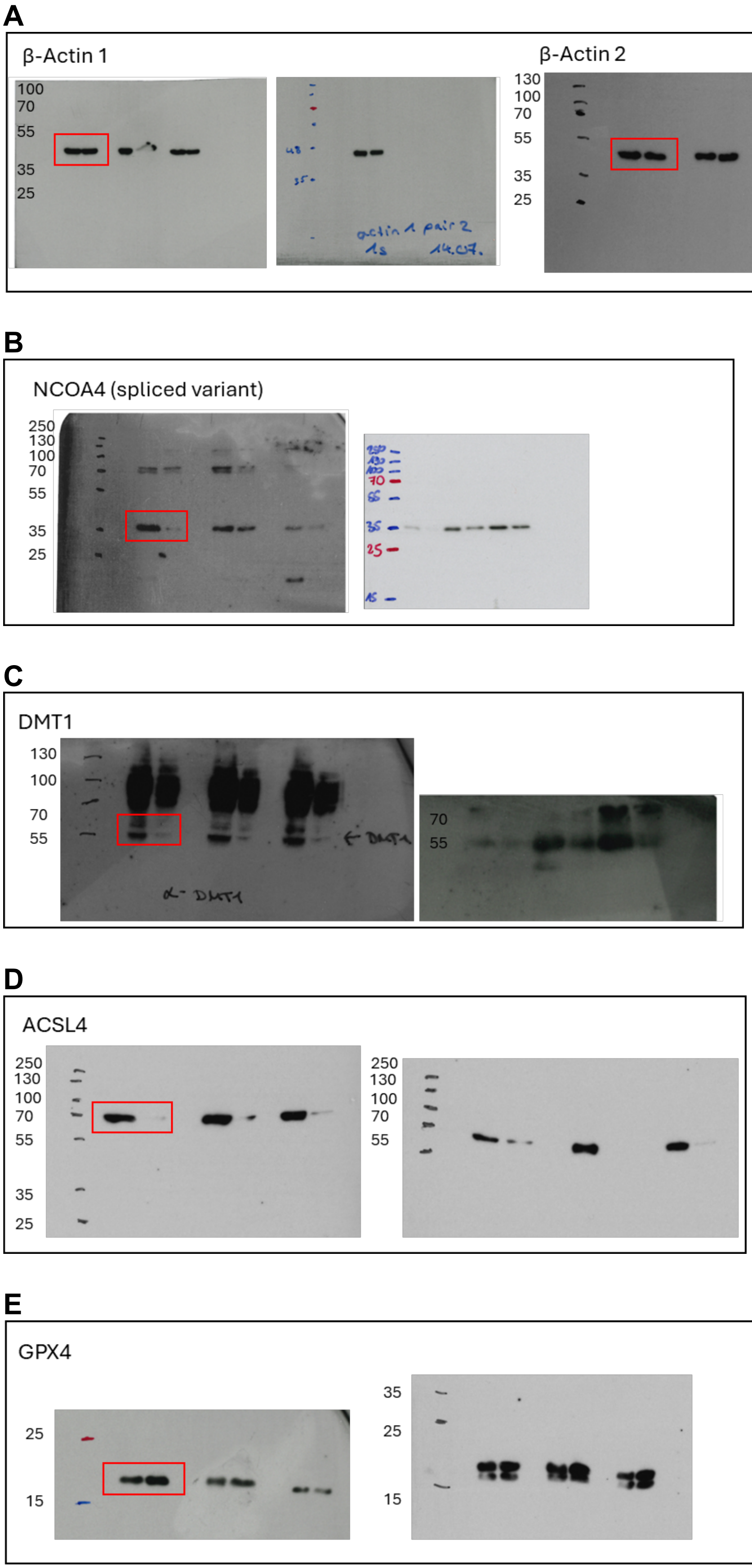

Supplementary Figure 6: Raw Western Blot analysis of miR-940 transfected cells
A,B,C,D,E) Western Blot analysis of miR-940-overexpressing HT-1080 cells with staining against β-Actin (A), NCOA4 spliced variant (B), DMT1 (C), ACSL4 (D) and GPX4 (E), PageRuler Plus Prestained Protein Ladder was used (Thermo Fisher Scientific)
