# Supplementary material for: MiR‐940 Suppresses Ferroptosis by Controlling Expression of Key Regulatory Genes
Source: Adv Sci (Weinh). 2026 May 29:e75830. Online ahead of print. doi: 10.1002/advs.75830 (PMC13336029; doi:10.1002/advs.75830)
Supplement: Supplementary file 8 — Supporting File 8: advs75830‐sup‐0008‐Legends for Supplementary Tables.docx. [file ADVS-9999-e75830-s007.docx]

**Legends for Supplementary Tables**

**Supplementary Table 1**

Predicted miR-940 binding sites of the ferroptosis regulators NCOA4, ACSL4, LPCAT3, DMT1, and GPX4 by databases TargetScan and miRDB. Includes the site type, site count, site position, score and score percentile.

**Supplementary Table 2**

Table of primers used in qRT-PCR. Includes primers for expression of ACSL4, DMT1, GPX4, LPCAT3, miR-94 and RNA Pol II.

**Supplementary Table 3**

Differentially regulated miRNAs identified by the GeCKO v2 whole genome CRISPR knockout screen. Sheet 1 includes the whole data set of the CRISPR screen. Sheet 2 shows 15 miRNAs that were significantly enriched and 15 miRNAs that were significantly depleted.

**Supplementary Table 4**

Raw counts from strand-specific RNA sequencing of total RNA isolated from pre-miR-940- and control-transfected cells, generated using Illumina NovaSeq technology.

**Supplementary Table 5**

Differential expression analysis of the RNA-sequencing data. Analysis was performed using the DESeq2 package in R (version 4.2.0). P-values were estimated using the Wald test and adjusted for multiple testing using the Benjamini–Hochberg method. Associations were considered significant at a false discovery rate (FDR) of 1% and an absolute log₂ fold change (|log₂FC|) > 1.

**Supplementary Table 6**

Table of lipidomics data with statistical analysis in Microsoft Excel (version 16.100). The peak areas of the respective lipid features were log2 transformed and scaled relative to the mean values of all samples. A two-tailed t-test was performed to select significant (p ≤ 0.05) lipid changes.
